# Structures of Cyclic Organosulfur Compounds From Garlic (*Allium sativum* L.) Leaves

**DOI:** 10.3389/fchem.2020.00282

**Published:** 2020-04-30

**Authors:** Masashi Fukaya, Seikou Nakamura, Hitoshi Hayashida, Daisuke Noguchi, Souichi Nakashima, Taichi Yoneda, Hisashi Matsuda

**Affiliations:** Department of Pharmacognosy, Kyoto Pharmaceutical University, Kyoto, Japan

**Keywords:** *Allium sativum* L., garlic, organosulfur compound, foliogarlic disulfane, foliogarlic trisulfane

## Abstract

Five new cyclic organosulfur compounds, foliogarlic disulfanes A_1_ (**1**), A_2_ (**2**), and A_3_ (**3**) and foliogarlic trisulfane A_1_ (**4**) and A_2_ (**5**), were isolated from the leaves of *Allium sativum* (garlic). The chemical structures of these compounds were elucidated on the basis of physicochemical evidence including Nuclear Magnetic Resonance (NMR) and Mass Spectrometry (MS). Compounds **1**–**5** were obtained as complex compounds with disulfane or trisulfane and tetrahydro-2*H*-difuro[3,2-*b*:2′,3′-*c*]furan-5(5a*H*)-one. In addition, the hypothetical biosynthetic pathways of these compounds were suggested.

## Introduction

*Allium* plants (Allieae), such as garlic, onion, and chives, have been cultivated as not only foodstuffs but also medicinal plants in the worldwide from ancient. For example, the extract of *Allium* plants, such as garlic, has shown anticancer, antidiabetic, and antibacterial effects. In addition, the National Cancer Institute in the United States had focused on *Allium* species as expecting cancer prevention (Theisen, 2001). *Allium* plants are well-known to have various cysteine sulfoxide derivatives such as alliin, methiin, and propiin (Rose et al., 2005). The type and contents of cysteine sulfoxides were also known to be different among *Allium* species (Fritsch and Keusgen, 2006). The cysteine sulfoxides change to thiosulfinates, such as allicin (Cavallito and Bailey, 1944; Cavallito et al., 1944), by the reaction with enzyme called alliinase (Ellmore and Feldberg, 1994) when the tissues of *Allium* plants are broken. Allicin has been reported to have several biological effects (Gebhardt et al., 1994; Briggs et al., 2000; Cañizares et al., 2004; Oommen et al., 2004; Arditti et al., 2005). However, unstable thiosulfinates including allicin are changed to organosulfur compounds, such as ajoene (Block et al., 1984), methyl 1-(methylthio)ethyl disulfane, and 5,7-diethyl-1,2,3,4,6-pentathiepane (Kuo et al., 1990). These compounds are also comparatively unstable and volatile. Although ajoene was known to have significantly anticancer effect, the application as medicines is difficult. On the other hand, several cyclic organosulfur compounds with anticancer effects isolated from the bulbs of the *Allium sativum* (garlic) have been reported by Nohara et al. (2012, 2013, 2014). Thus, cyclic organosulfur compounds are important on the development of medicines including anticancer effect. On the basis of this background, we have isolated several comparatively stable organosulfur compounds from *Allium fistulosum* (green onion and welsh onion) (Fukaya et al., 2018, 2019a) and *Allium schoenoprasum* var. *foliosum* (Japanese chive) (Fukaya et al., 2019b). In the course of our ongoing research program for discovery of bioactive organosulfur compounds, the constituents from the leaves of *Allium sativum* were examined. In this article, we discuss the isolation and the structure elucidation of cyclic organosulfur compounds, foliogarlic disulfanes A_1_ (**1**), A_2_ (**2**), and A_3_ (**3**) and foliogarlic trisulfane A_1_(**4**) and A_2_ (**5**), from the leaves of *A. sativum* and the biosynthetic pathways.

## Results and Discussions

**Figure 1:**
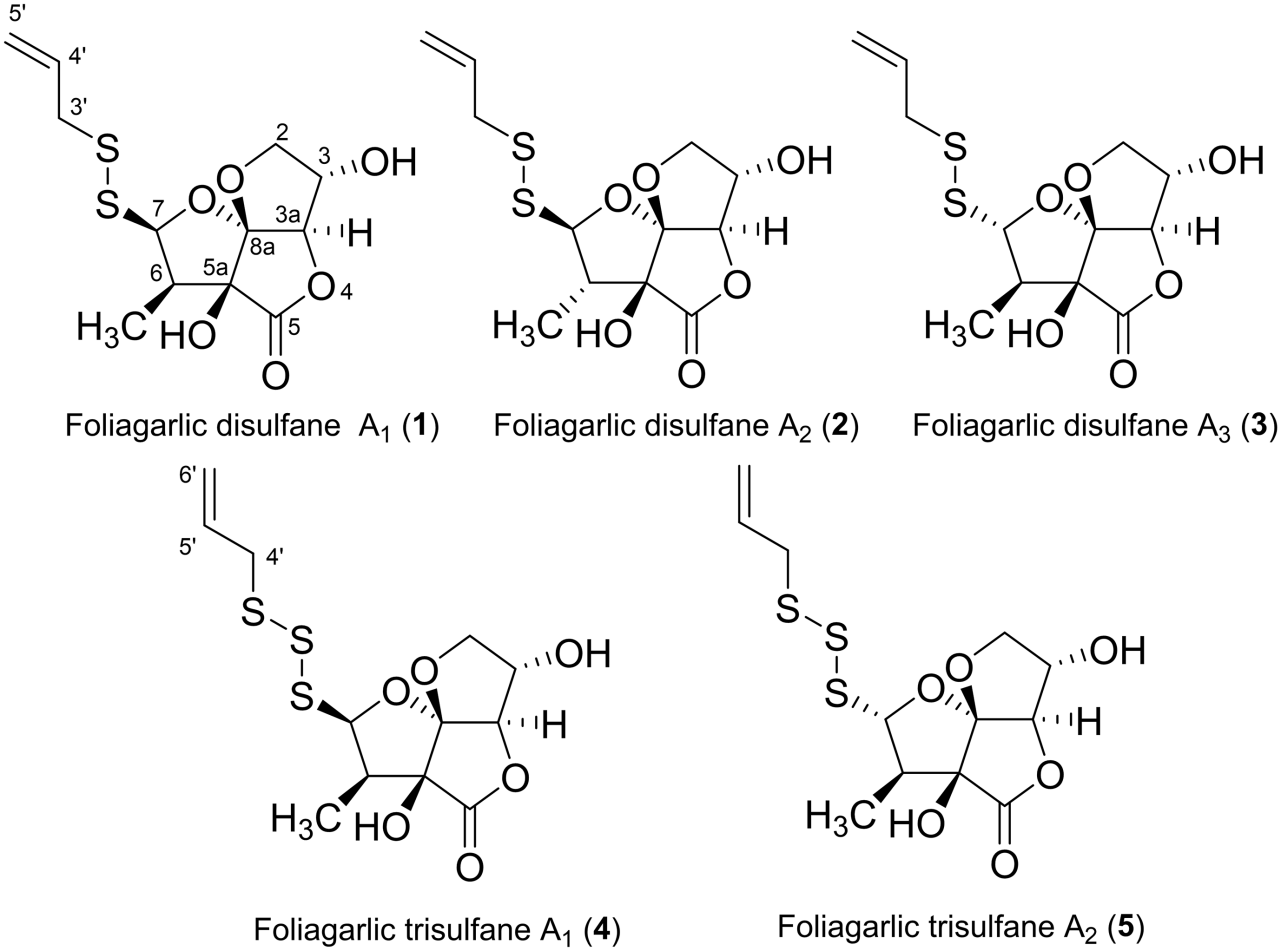
Structures of new compounds **1-5** from the leaves of *A. sativum*.

The fresh leaves of *A. sativum* (15.0 kg) were mixed with water. Then, acetone was added into the mixture to be 80% acetone solution. The solution was concentrated after standing for 4 days (96 h) at room temperature. The acetone extract was portioned between ethyl acetate (EtOAc) and water. The organic fraction was evaporated *in vacuo* and obtained EtOAc fraction as syrup (41.98 g, 0.27% from the plant). The EtOAc fraction was also subjected with the normal and reversed-phase column chromatography and high-performance liquid chromatography (HPLC) to give foliogarlic disulfanes A_1_ (**1**, 0.00013%), A_2_ (**2**, 0.00021%), and A_3_ (**3**, 0.00009%) and foliogarlic trisulfanes A_1_ (**4**, 0.00015%) and A_2_ (**5**, 0.00008%) (Figure 1).

**Figure 2:**
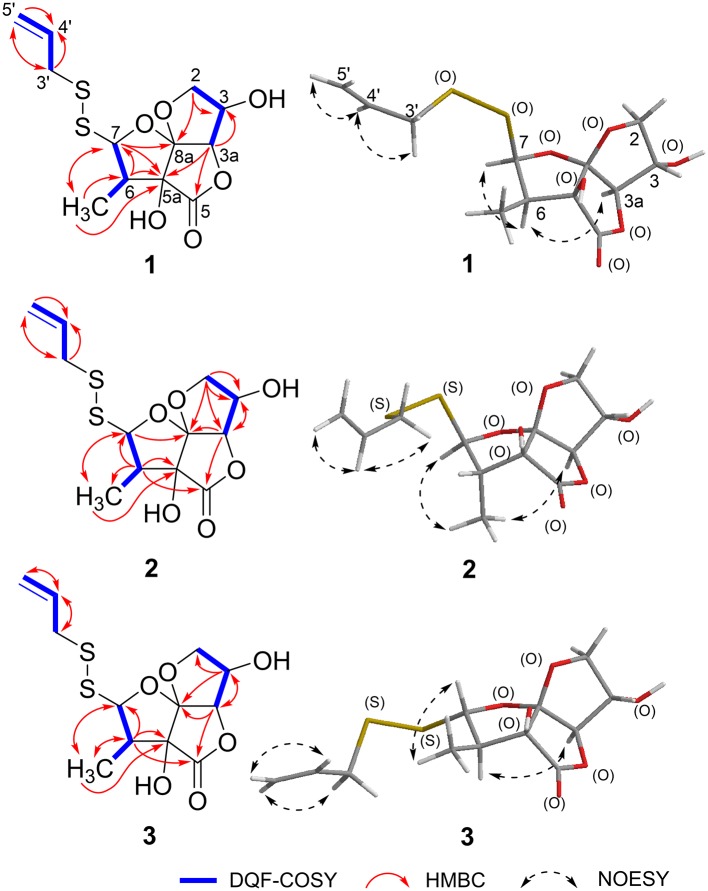
Key-correlations of 2D NMR and NOESY of **1-3**.

Foliogarlic disulfanes A_1_ (**1**) was obtained as yellow oil and showed positive optical rotation (+160.9). In the Electrospray Ionization MS (ESIMS) measurement of **1**, a pseudomolecular ion peak [M + Na]^+^ was observed at *m/z* 343.0282, and the molecular formula was determined as C_12_H_16_O_6_S_2_ on the basis of the High Resolution ESIMS (HRESIMS) peak and the ^13^C NMR data. The ^13^C NMR spectra of **1** showed signals corresponding to a secondary methyl group at δ_C_ 9.1 (6-CH_3_), a methine at δ_C_ 48.1 (C-6), a diastereotopic oxygen-bearing methylene at δ_C_ 74.98 (C-3); an oxygen-bearing methine at δ_C_ 75.03 (C-2); two methines neighboring the electron-withdrawing atom at δ_C_ 90.4 (C-3a) and δ_C_ 103.2 (C-7); an oxygen-bearing quaternary carbon at δ_C_ 79.6 (C-5a); a two-oxygen–bearing quaternary carbon at δ_C_ 119.1 (C-8a); and a lactone carbonyl carbon at δ_C_ 175.4 (C-5) (Table 1, Figure 2, and Supplementary Material). The correlations of COSY double-quantum filter (DQF COSY) NMR spectroscopy were observed between 6-CH_3_, H-6, and H-7 and between H-2, H-3, and H-3a (Figure 2). The heteronuclear multiple-bond correlation (HMBC) spectrum of **1** is shown in Figure 2. Namely, the correlation of H-2 to C-8a, H-7 to C-5a and H-3a to C-5a indicates acetal structure, the correlation of H-3a to C-5 and C-5a indicates a lactone, and the correlations of H-6 to C-5a and C-7, H-7 to C-5 and 6-CH_3_, 6-CH_3_ to C-5a, C-6, and C-7 indicate a secondary methyl moiety. These evidences indicate that compound **1** had a tetrahydro-2*H*-difuro[3,2-b:2′,3′-c]furan-5(5a*H*)-one skeleton. In addition, 1-propenyl disulfane structure at the side chain was confirmed by High Resolution MS (HRMS) and NMR (Nuclear Magnetic Resonance) data. Next, the NOESY spectrum of **1** showed key correlations between H-3a and H-6; and H-6 and H-7 (Figure 2). The results prove that the relative configurations among H-3, H-6, and H-7 were of the same orientation, respectively. Furthermore, the ^1^H and ^13^C NMR signals of **1** assigned to tetrahydro-2*H*-difuro[3,2-b:2′,3′-c]furan-5(5a*H*)-one skeleton were superimposable on those of known compound, kujounin A_3_, except for 1-propenyl disulfane moiety (Fukaya et al., 2019a). All the evidences support that the chemical structure of **1** was (3S^*^,3aR^*^,5aS^*^,6R^*^,7R^*^,8aR^*^)-3,5a-dihydroxy-6-methyl-7-(allyldisulfanyl)tetrahydro-2*H*-difuro[3,2-*b*:2′,3′-*c*]furan-5(5a*H*)-one.

**Table 1:** ^1^H NMR and ^13^C NMR data of **1** and **2**.

**Position**	**1**		**2**		**2**	
	***δ_*H*_* (*J*, Hz)^**a**^**	***δ_*C*_*^**a**^**	***δ_*H*_* (*J*, Hz)^**a**^**	***δ_*C*_*^**a**^**	***δ_*H*_* (*J*, Hz)^**b**^**	***δ_*C*_*^**b**^**
2	4.05 (m)	75.03	4.00 (dd, *J* = 5.5, 7.5)	75.0	α 4.11 (dd, *J* = 4.8, 10.3)	74.2
			4.01 (dd, *J* = 5.5, 9.5)		β 4.23 (dd, *J* = 3.4, 10.3)	
3	4.30 (m)	74.98	4.30 (m)	75.5	4.44 (m)	74.4
3a	4.62 (d-like)	90.4	4.60 (d, *J* = 2.5)	90.4	4.64 (d, *J* = 1.3)	88.6
5		175.4		175.0		171.4
5a		79.6		82.3		81.6
6	2.91 (m)	48.1	2.63 (m)	49.8	2.75 (m)	49.7
7	5.55 (d, 7.0)	103.2	4.78 (d, *J* = 7.0)	94.5	5.00 (d, *J* = 3.5)	97.5
8a		119.1		117.0		117.0
3′	3.45 (m)	43.5	3.47 (d, *J* = 7.5)	43.8	3.48 (m)	42.7
4′	5.88 (m)	134.6	5.86 (m)	134.4	5.88 (m)	133.0
5′	5.09 (d like, 10.0)	118.9	5.12 (d-like, *J* = 9.5)	119.1	5.15 (d-like, *J* = 11.6)	120.0
	5.19 (d like, 17.0)		5.18 (d-like, *J* = 16.0)		5.20 (d-like, *J* = 16.4)	
6-CH_3_	1.18 (d, 7.0)	9.1	1.13 (d, *J* = 7.5)	12.6	1.21 (d, *J* = 7.6)	14.0

a *^1^H NMR, ^13^C NMR (CD_3_OD, 500 MHz)*.

b *^1^H NMR, ^13^C NMR (CDCl_3_, 600 NMR)*.

Foliogarlic disulfanes A_2_ (**2**) and A_3_ (**3**) were isolated as yellow oil with positive specific rotations (**2**: ${\left[\alpha \right]}_{\text{D}}^{25}$ + 139.0° in MeOH) and negative specific rotations (**3**: ${\left[\alpha \right]}_{\text{D}}^{25}$ – 213.6° in MeOH). In the ESIMS spectra of **2** and **3**, the same quasi-molecular ion peaks (**2** and **3**: [M+Na]^+^) were observed at *m*/*z* 343. The molecular formulas (**2** and **3**: C_12_H_16_O_6_S_2_) were determined on the basis of HRESIMS peaks at [**2**: *m/z* 343.0277, **3**: *m/z* 343.0282 (calcd. 343.0281)] and the ^13^C NMR data. The ^1^H and ^13^C NMR spectrum of **2** and **3** showed signals corresponding to a secondary methyl group, a methine, a diastereotopic oxygen-bearing methylene, and an oxygen-bearing methine (Tables 1, 2 and Figure 2). On the basis of this evidence and detailed examination of DQF COSY and HMBC experiments, the planner structures of **2** and **3** were found to be the same as that of **1**. Next, the relative configurations of **2** and **3** were characterized by the detailed NOESY experiments. The NOESY spectrum of **2** showed key correlations between H-3a and 6-CH_3_; and H-7 and 6-CH_3_ (Figure 2). The NOESY spectrum of **3** showed key correlations between H-3a and H-6; and H-7 and 6-CH_3_ (Figure 2). In addition, the ^1^H and ^13^C NMR signals of **2** and **3** were superimposable on those of known compounds, kujounin A_1_ and A_2_, respectively, except for 1-propenyl disulfane structure (Fukaya et al., 2018). Consequently, the chemical structures of foliogarlic disulfanes A_2_ (**2**) and A_3_ (**3**) were determined as (3S^*^,3aR^*^,5aS^*^,6S^*^,7R^*^,8aR^*^)-3,5a-dihydroxy-6-methyl-7-(allyldisulfanyl)tetrahydro-2*H*-difuro[3,2-*b*:2′,3′-*c*]furan-5(5a*H*)-one and (3S^*^,3aR^*^,5aS^*^,6R^*^,7S^*^,8aR^*^)-3,5a-dihydroxy-6-methyl-7-(allyldisulfanyl)tetrahydro-2*H*-difuro[3,2-*b*:2′,3′-*c*]furan-5(5a*H*)-one, respectively.

**Table 2:** ^1^H NMR and ^13^C NMR data of **3**.

**Position**	**3**			
	***δ_*H*_* (*J*, Hz)^**a**^**	***δ_*C*_*^**a**^**	***δ_*H*_* (*J*, Hz)^**b**^**	***δ_*C*_*^**b**^**
2	4.04 (dd, *J* = 4.5, 10.0)	75.9	α 4.11 (dd, *J* = 4.1, 10.3)	75.4
	4.07 (dd, *J* = 3.0, 10.0)		β 4.27 (dd, *J* = 1.4, 10.3)	
3	4.29 (m)	74.7	4.48 (m)	73.9
3a	4.75 (s-like)	89.9	4.85 (s-like)	87.5
5		174.9		172.2
5a		80.0		78.4
6	2.69 (m)	47.9	2.71 (m)	46.5
7	5.11 (d, *J* = 10.0)	96.3	5.16 (d, *J* = 9.6)	95.5
8a		119.1		117.2
3′	3.46 (d, *J* = 7.5)	44.1	3.44 (d, *J* = 7.6)	43.2
4′	5.85 (m)	134.3	5.85 (m)	132.5
5′	5.14 (d like, *J* = 10.0)	119.4	5.19 (d like, *J* = 10.3)	119.5
	5.20 (d like, *J* = 16.5)		5.22 (d like, *J* = 15.8)	
6-CH_3_	1.09 (d, *J* = 6.5)	8.4	1.19 (d, *J* = 6.8)	7.9

a *^1^H NMR, ^13^C NMR (CD_3_OD, 500 MHz)*.

b *^1^H NMR, ^13^C NMR (CDCl_3_, 600 NMR)*.

**Figure 3:**
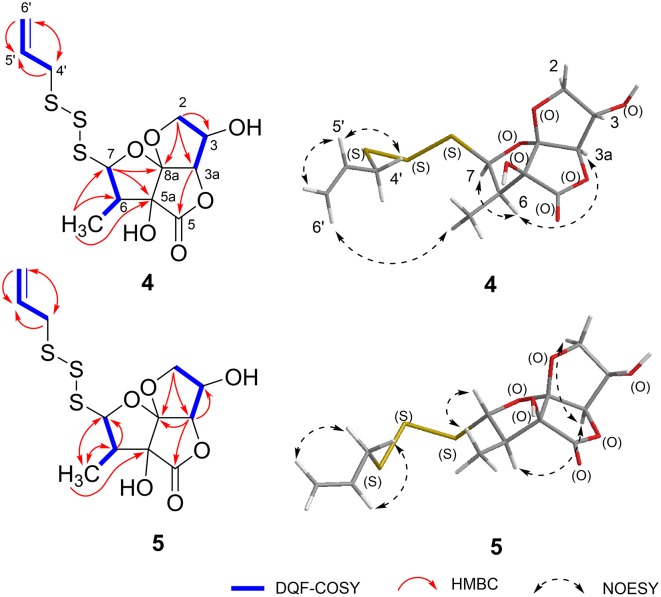
Key-correlations of 2D NMR and NOESY of **4** and **5**.

Foliogarlic trisulfanes A_1_ (**4**) and A_2_ (**5**) were isolated as yellow oil with positive specific rotations (**4**: ${\left[\alpha \right]}_{\text{D}}^{25}$ +124.6° in MeOH) and negative specific rotations (**5**: ${\left[\alpha \right]}_{\text{D}}^{25}$ −119.8° in MeOH). In the ESIMS spectra of **4** and **5**, the same quasi-molecular ion peaks (**4** and **5**: [M+Na]^+^) were observed at *m*/*z* 375. The molecular formulas (**4** and **5**: C_12_H_16_O_6_S_3_) were determined on the basis of HRESIMS peaks at [**4**: *m/z* 374.9998, **3**: *m/z* 374.0003 (calcd. 374.0001)], and the ^13^C NMR data. On the basis of the detailed analysis of the ^1^H and ^13^C NMR, 2D-NMR (DQF COSY, HMBC, NOESY) spectrum of **4** and **5**, the relative structures of tetrahydro-2*H*-difuro[3,2-*b*:2′,3′-*c*]furan-5(5a*H*)-one skeleton on **4** and **5** were found to be the same as those of **1** and **3**, respectively (Tables 3, 4 and Figure 3). Next, the ^1^H and ^13^C NMR spectrum at the side chain showed signals corresponding to an allyl group, as well as those of compounds **1**–**3**. The determination of the sulfur linkage was confirmed by the HRMS spectrum. Namely, the pseudomolecular formula was established as C_12_H_16_O_6_S_3_Na. Therefore, compounds **4** and **5** were found to have a trisulfane bridge. Finally, the relative configurations of **4** and **5** were characterized by the comparison of ^13^C NMR data with **1** and **3** and the NOESY experiments. The ^13^C NMR signals of **4** and **5** were superimposable on those of **1** and **3**. All the evidences supported that the chemical structures of **4** and **5** were (3S^*^,3aR^*^,5aS^*^,6R^*^,7R^*^,8aR^*^)-3,5a-dihydroxy-6-methyl-7-(allyltrisulfanyl)tetrahydro-2*H*-difuro[3,2-*b*:2′,3′-*c*]furan-5(5a*H*)-one and (3S^*^,3aR^*^,5aS^*^,6R^*^,7S^*^,8aR^*^)-3,5a-dihydroxy-6-methyl-7-(allyltrisulfanyl)tetrahydro-2*H*-difuro[3,2-*b*:2′,3′-*c*]furan-5(5a*H*)-one, respectively.

**Table 3:** ^1^H NMR and ^13^C NMR data of **4**.

**Position**	**4**			
	***δ_*H*_* (*J*, Hz)^**a**^**	***δ_*C*_*^**a**^**	***δ_*H*_* (*J*, Hz)^**b**^**	***δ_*C*_*^**b**^**
2	4.07 (m)	75.7	4.28 (m)	75.0
3	4.26 (m)	75.0	4.42 (m)	73.9
3a	4.66 (d, *J* = 1.5)	90.6	4.68 (s-like)	88.5
5		175.1		170.2
5a		79.5		78.0
6	2.97 (m)	48.0	2.83 (m)	47.0
7	5.68 (d, *J* = 7.0)	102.3	5.71 (d, *J* = 6.9)	99.5
8a		119.1		119.0
3′	3.59 (dd, *J* = 6.5, 13.0)	42.3	3.49 (m)	42.6
	3.63 (dd, *J* = 7.5, 13.0)			
4′	5.86 (m)	134.3	5.86 (m)	132.5
5′	5.17 (d-like, *J* = 10.0)	119.5	5.16 (d-like, *J* = 9.6)	119.6
	5.22 (d-like, *J* = 17.0)		5.21 (*J* = 16.5)	
6-CH_3_	1.16 (d, *J* = 7.0)	9.2	1.28 (d, *J* = 6.9)	8.6

a *^1^H NMR, ^13^C NMR (CD_3_OD, 500 MHz)*.

b *^1^H NMR, ^13^C NMR (CDCl_3_, 600 NMR)*.

**Table 4:** ^1^H NMR and ^13^C NMR data of **5**.

**Position**	**5**	
	***δ_*H*_* (*J*, Hz)^**a**^**	***δ_*C*_*^*a*^**
2	4.02 (m)	75.6
3	4.25 (m)	75.1
3a	4.71 (d, *J* = 1.5)	90.2
5		175.0
5a		80.1
6	2.63 (m)	48.2
7	5.23 (d, *J* = 9.5)	96.6
8a		118.8
3′	3.56 (m)	42.7
4′	5.82 (m)	134.0
5′	5.15 (d like, *J* = 10.0)	119.9
	5.21 (d like, *J* = 16.5)	
6-CH_3_	1.11 (d, *J* = 6.5)	8.7

a *^1^H NMR, ^13^C NMR (CD_3_OD, 500 MHz)*.

**Figure 4:**
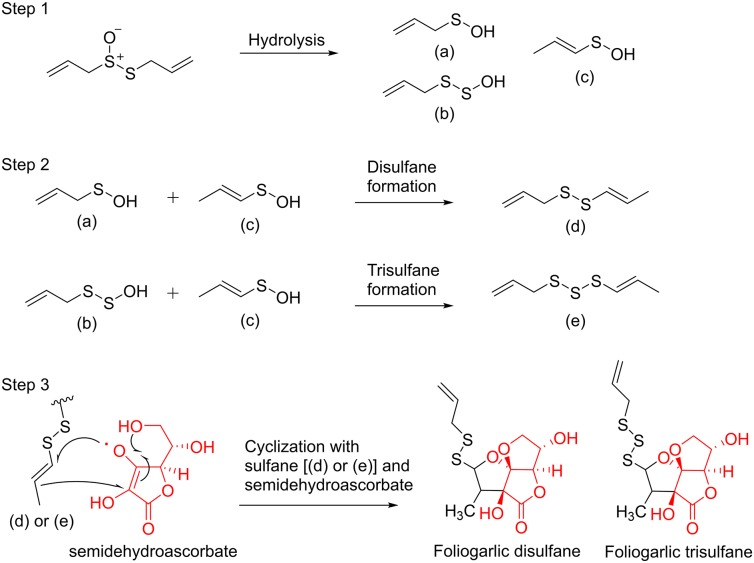
The biological synthetic pathway for compounds **1-5**.

The biological synthetic pathways for compounds **1**–**5** are presumed. At first, allicin is generated from alliin by alliinase when plant tissues of *A. sativum* are broken. Next, allicin is decomposed into intermediates (a), (b), and (c) by hydrolysis and is reconstructed to disulfane (d) and trisulfane (e) (Jacob, 2006). Finally, the structure of tetrahydro-2*H*-difuro[3,2-*b*:2′,3′-*c*]furan-5(5a*H*)-one skeleton is formed from semidehydroascorbate by cyclization and sulfane formation with the intermediates d and e. Consequently, compounds **1**–**5** were presumed to be obtained (Figure 4).

## Conclusion

Five new organosulfur compounds, foliogarlic disulfanes **1**–**3** and foliogarlic trisulfanes **4** and **5**, were isolated from the leaves of *A. sativum*. These compounds **1**–**5** have a tetrahydro-2*H*-difuro[3,2-*b*:2′,3′-*c*]furan-5(5a*H*)-one skeleton with methyl group at 6-position and 2-propenyl disulfane or 2-propenyl trisulfane group at 7-position. Particularly, foliogarlic trisulfanes **4** and **5** with a trisulfane moiety are a rare compound derived from medicinal plants. The biological effects of these cyclic organosulfur compounds should be studied further.

## Experimental

### General

The following instruments were used to obtain physical data: specific rotations, a Horiba (Kyoto, Japan) SEPA-300 digital polarimeter (*l* = 5 cm); IR spectra, JASCO (Tokyo, Japan) FT/IR-4600 Fourier Transform Infrared Spectrometer; ESIMS, Agilent Technologies (CA, US) Quadrupole LC/MS 6130; HRESIMS, SHIMADZU LCMS-IT-TOF; ^1^H NMR spectra, JEOL (Tokyo, Japan) JNM-LA 500 (500 MHz) spectrometer; ^13^C-NMR spectra, JEOL JNM-LA 500 (125 MHz) spectrometer; NOESY spectra, JNM-ECA 600 (600 MHz) spectrometer; HPLC, a Shimadzu (Kyoto, Japan) SPD-20AVP UV-VIS detector. YMC-triart C18 (250 × 4.6 mm i.d. and 250 × 10 mm i.d.) and YMC-triart PFP (250 × 4.6 mm i.d. and 250 × 10 mm i.d.) columns were used for analytical and preparative purposes. The following experimental materials were used for chromatography: normal-phase silica gel column chromatography, silica gel BW-200 (Fuji Silysia Chemical, Ltd. (Aichi, Japan, 150–350 mesh); reversed-phase silica gel column chromatography, Cosmosil 140C_18_-OPN [Nacalai Tesque (Kyoto, Japan)], TLC, precoated TLC plates with silica gel 60F_254_ [Merck (NJ, US), 0.25 mm] (ordinary phase), and silica gel RP-18 F_254S_ (Merck, 0.25 mm) (reversed phase); reversed-phase HPTLC, precoated TLC plates with silica gel RP-18 WF_254S_. Detection was achieved by spraying with 1% Ce (SO_4_) 2–10% aqueous H_2_SO_4_ followed by heating.

### Plant Material

Fresh leaves of *A. sativum* cultivated in Kochi prefecture, Japan, were obtained as commercial products purchased from Japan Agricultural Cooperatives (JA) farmers' market (Kochi, Japan) in April 2017. The plants were identified by the authors (H.M. and S.N.).

### Extraction and Isolation

The fresh leaves of *A. sativum* (15.2 kg) were chopped and mixed with water, and then acetone was added to the mixture to be 80% acetone solution. The mixture was soaked for 4 days (96 h) at room temperature. Evaporation of the filtrate under reduced pressure provided acetone extract (1,500.37 g, 9.87%). The extract was partitioned between EtOAc and H_2_O (1:1, vol/vol) to obtain EtOAc fraction (41.98 g, 0.27%) and aqueous phase. The EtOAc-soluble fraction (41.98 g) was subjected to normal phase silica gel column chromatography [1,260 g, CHCl_3_-MeOH (1:0 → 100:1 → 50:1 → 30:1 → 10:1 → 0:1, vol/vol)] to give nine fractions {Fr.1 (1,471.6 mg), Fr.2 (715.5 mg), Fr.3 (7,193.2 mg), Fr.4 (8,339.2 mg), Fr.5 (4,085.8 mg), Fr.6 (1,334.9 mg), Fr.7 (4,367.0 mg), Fr.8 (617.7 mg), Fr.9 (5,841.2 mg)}. r. 5 (4,085.8 mg) was further separated by reversed-phase silica gel column chromatography [200 g, MeOH-H_2_O (2:8 → 4:6 → 6:4 → 8:2 → 1:0, vol/vol)] to give 13 fractions {Fr.5-1 (52.3 mg), Fr.5-2 (21.0 mg), Fr.5-3 (31.0 mg), Fr.5-4 (19.1 mg), Fr.5-5 (84.4 mg), Fr.5-6 (281.7 mg), Fr.5-7 (43.5 mg), Fr.5-8 (26.3 mg), Fr.5-9 (67.9 mg), Fr.5-10 (482.9 mg), Fr.5-11 (2,637.7 mg), Fr.5-12 (178.7 mg), Fr.5-13 (16.9 mg)}. Fr.5-5 (84.4 mg) was purified by HPLC {mobile phase: MeOH-H_2_O (35:65, vol/vol) [YMC-triart PFP (250 × 10 mm i.d.)]} to give **1** (6.0 mg) and **2** (16.3 mg). Fr.5-6 (281.7 mg) was purified by HPLC {mobile phase: MeOH-H_2_O (50:50, vol/vol) [YMC-triart C18 (250 × 10 mm i.d.)]} to give **1** (14.6 mg), **2** (16.0 mg), **4** (24.2 mg), and **5** (12.3 mg). Fr.5-6-5 (37.1 mg) was purified by HPLC {mobile phase: MeOH-H_2_O (45:55, vol/vol) [YMC-triart C18 (250 × 10 mm i.d.)]} to give **3** (14.7 mg) and **4** (4.5 mg).

### Foliogarlic Disulfane A_1_ (1)

Yellow oil; ${\left[\alpha \right]}_{\text{D}}^{25}$ +160.9 (MeOH); HRESIMS: calcd for C_12_H_16_O_6_S_2_Na (M+Na)^+^: 343.0281, found: 343.0282; IR(ATR): 3,400, 2,975, 1,782 cm^−1^; ^1^H NMR (CD_3_OD), ^13^C NMR (CD_3_OD, 500 MHz): given in Table 1.

### Foliogarlic Disulfane A_2_ (2)

Yellow oil; ${\left[\alpha \right]}_{\text{D}}^{25}$ +139.0 (MeOH); HRESIMS: calcd for C_12_H_16_O_6_S_2_Na (M+Na)^+^: 343.0281, found: 343.0277; IR(ATR): 3,400, 2,970, 1,785 cm^−1^; ^1^H NMR (CD_3_OD, CDCl_3_), ^13^C NMR (CD_3_OD, CDCl_3_): given in Table 1.

### Foliogarlic Disulfane A_3_ (3)

Yellow oil; ${\left[\alpha \right]}_{\text{D}}^{25}-$213.6 (MeOH); HRESIMS: calcd for C_12_H_16_O_6_S_2_Na (M+Na)^+^: 343.0281, found: 343.0282; IR(ATR): 3,400, 2,981, 1,785 cm^−1^; ^1^H NMR (CD_3_OD, CDCl_3_), ^13^C NMR (CD_3_OD, CDCl_3_): given in Table 2.

### Foliogarlic Trisulfane A_1_ (4)

Yellow oil; ${\left[\alpha \right]}_{\text{D}}^{25}$ +124.6 (MeOH); HRESIMS: calcd for C_12_H_16_O_6_S_3_Na (M+Na)^+^: 375.0001, found: 374.9998; IR(ATR): 3,400, 2,975, 1,780 cm^−1^; ^1^H NMR (CD_3_OD, CDCl_3_), ^13^C NMR (CD_3_OD, CDCl_3_): given in Table 3.

### Foliogarlic Trisulfane A_2_ (5)

Yellow oil; ${\left[\alpha \right]}_{\text{D}}^{25}-$119.8 (MeOH); HRESIMS: calcd for C_12_H_16_O_6_S_3_Na (M+Na)^+^: 375.0001, found: 375.0003; IR(ATR): 3,400, 2,931, 1,789 cm^−1^; ^1^H NMR (CD_3_OD), ^13^C NMR (CD_3_OD): given in Table 4.

## Data Availability Statement

All datasets generated for this study are included in the article/Supplementary Material.

## Author Contributions

MF: isolation of constituents and Structure elucidation of new compounds. SeiN: Structure elucidation of new compounds and overall supervision in this study. HH and DN: isolation of constituents. SouN: Preparation of plant extracts and overall supervision in this study. TY: Structure elucidation of new compounds. HM: overall supervision in this study.

## Conflict of Interest

The authors declare that the research was conducted in the absence of any commercial or financial relationships that could be construed as a potential conflict of interest.
