# Supplementary material for: Structures of Cyclic Organosulfur Compounds From Garlic (Allium sativum L.) Leaves
Source: Front Chem. 2020 Apr 30;8:282. doi: 10.3389/fchem.2020.00282 (PMC7205455; doi:10.3389/fchem.2020.00282)
Supplement: Supplementary file 1 [file Data_Sheet_1.PDF]

Supplementary materials

Structures of cyclic organosulfur compounds from garlic (*Allium sativum* L.) leaves

Masashi Fukaya[1], Seikou Nakamura[1], Hitoshi Hayashida[1], Daisuke Noguchi[1],

Souichi Nakashima[1], Taichi Yoneda[1], Hisashi Matsuda[1*]

Kyoto Pharmaceutical University, Misasagi, Yamashina-ku, Kyoto 607-8412, Japan

* Corresponding author. Tel.: +81 75 595 4633; Fax: +81 75 595 4768;

E-mail address: matsuda@mb.kyoto-phu.ac.jp

Contents

[1] ^{1}H -NMR, ^{13}C -NMR, NOESY, ESI-MS, and IR spectra of compound **1** …………..…S3-6

[2] ^{1}H -NMR, ^{13}C -NMR, NOESY, ESI-MS, and IR spectra of compound **2** …………..…S7-9

[3] ^{1}H -NMR, ^{13}C -NMR, NOESY, ESI-MS, and IR spectra of compound **3** …………..…S10-13

[4] ^{1}H -NMR, ^{13}C -NMR, NOESY, ESI-MS, and IR spectra of compound **4** …………..…S14-17

[5] ^{1}H -NMR, ^{13}C -NMR, NOESY, ESI-MS, and IR spectra of compound **5** …………..…S18-21

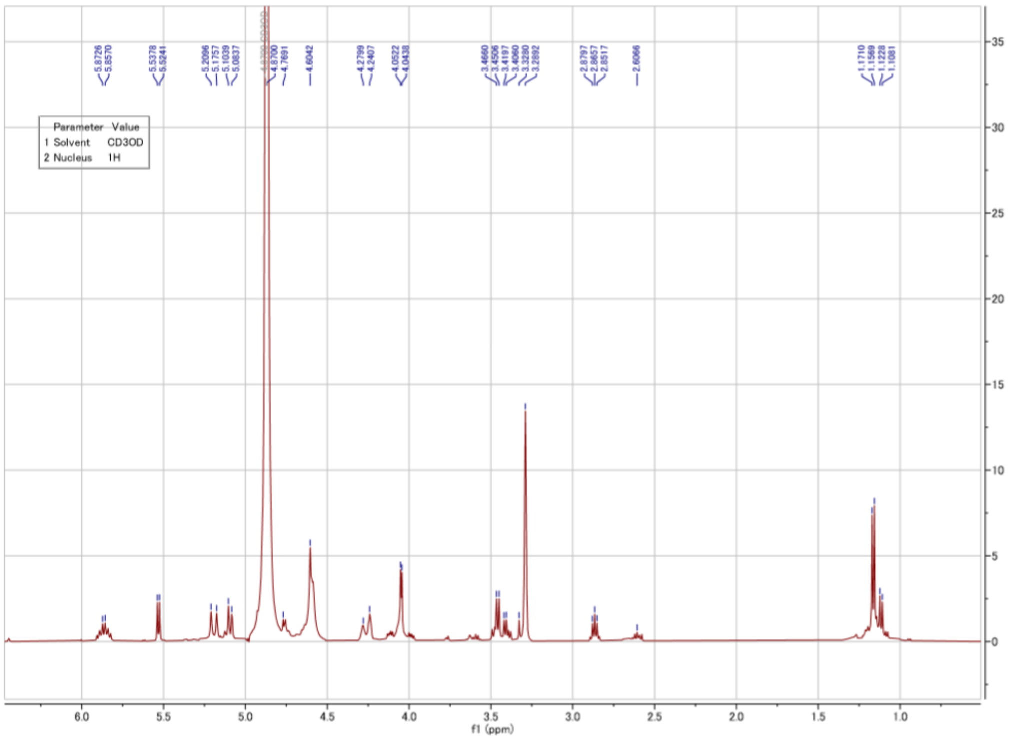

[1] H -NMR spectrum of compound **1**. Measured in CD_3OD.

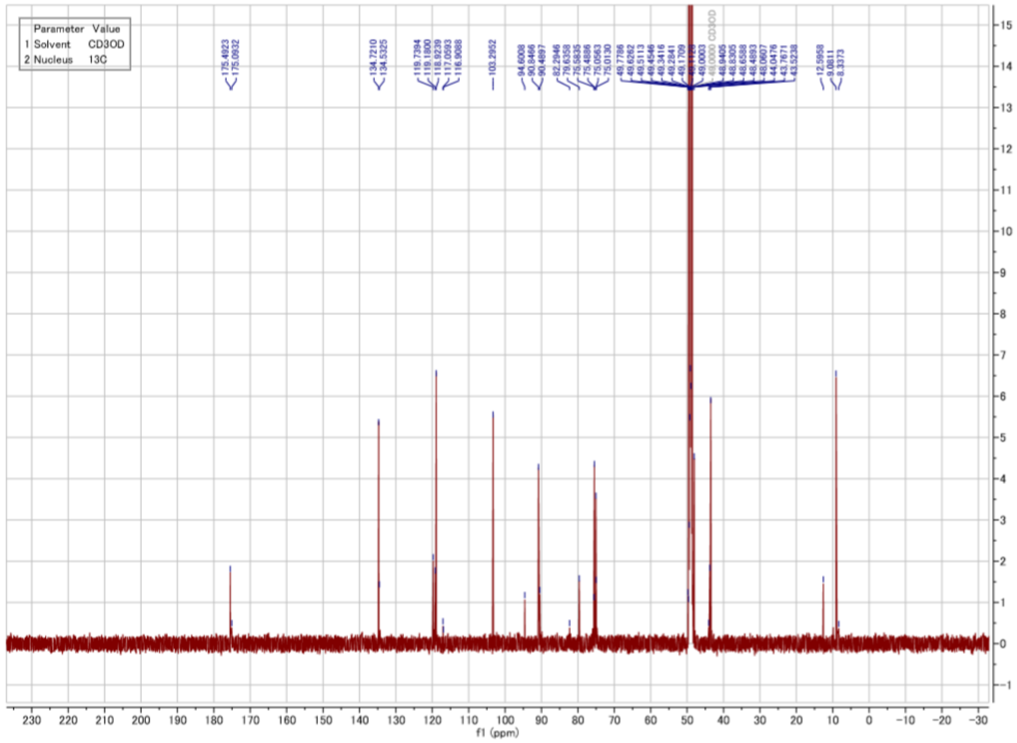

^{13}C -NMR spectrum of compound **1**. Measured in CD_3OD.

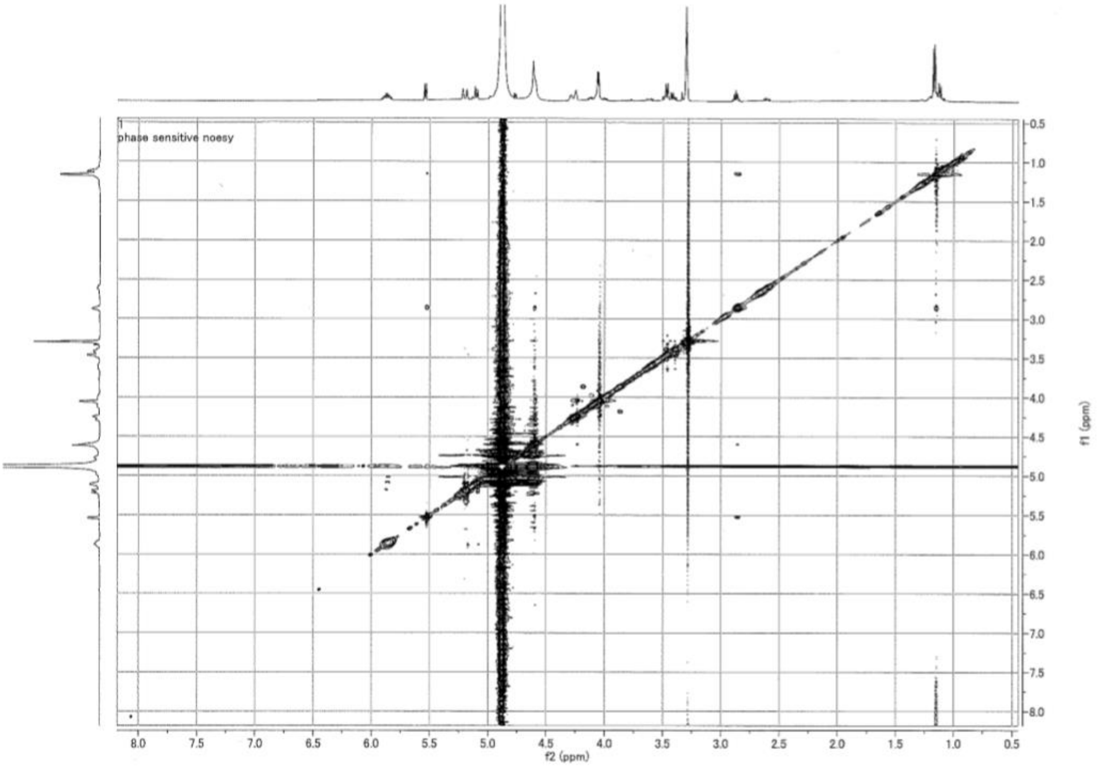
phase sensitive noesy
f2 (ppm)
f1 (ppm)

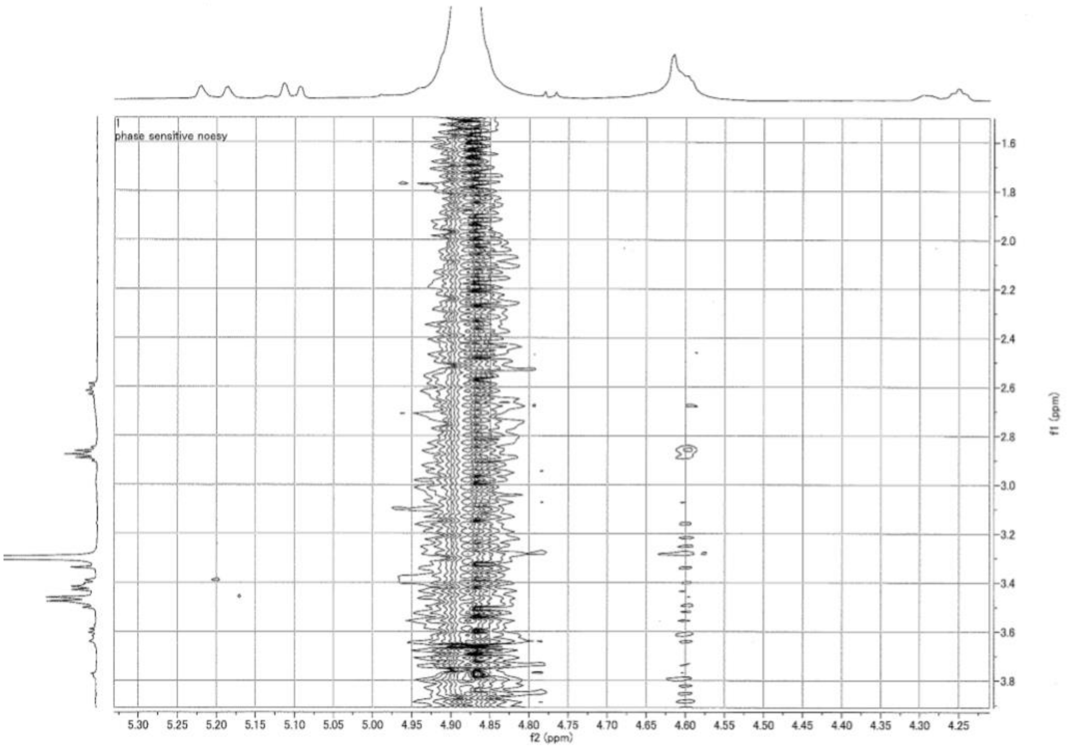
phase sensitive noesy
f2 (ppm)
f1 (ppm)

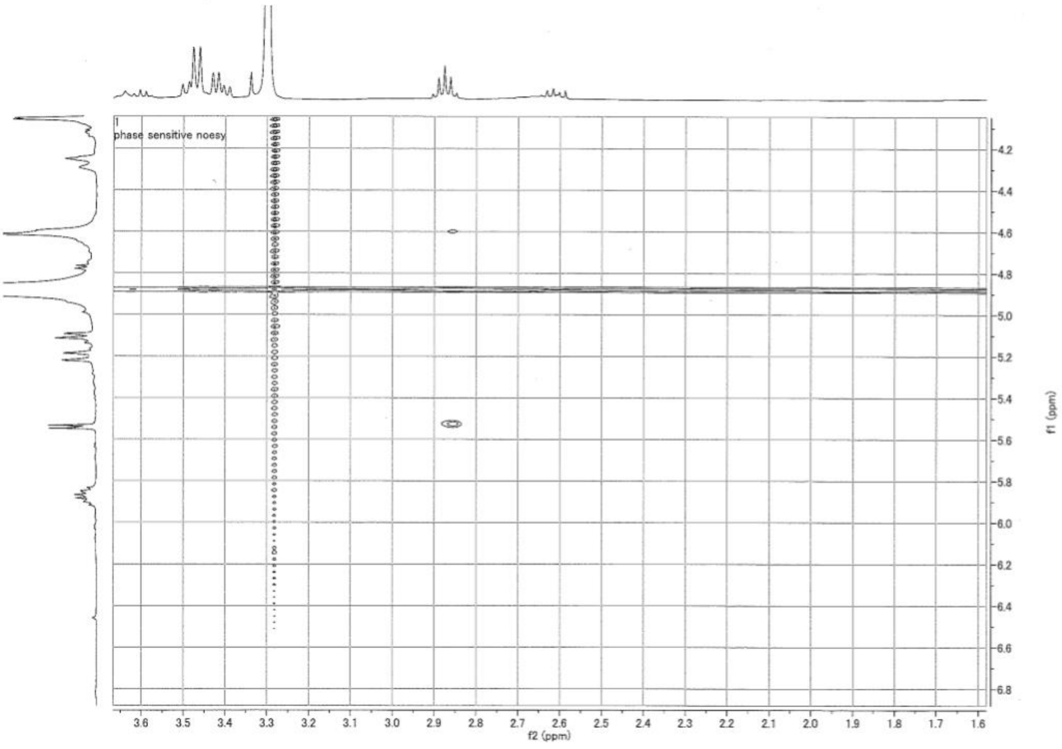

NOESY spectrum of compound **1**. Measured in CD_3OD.

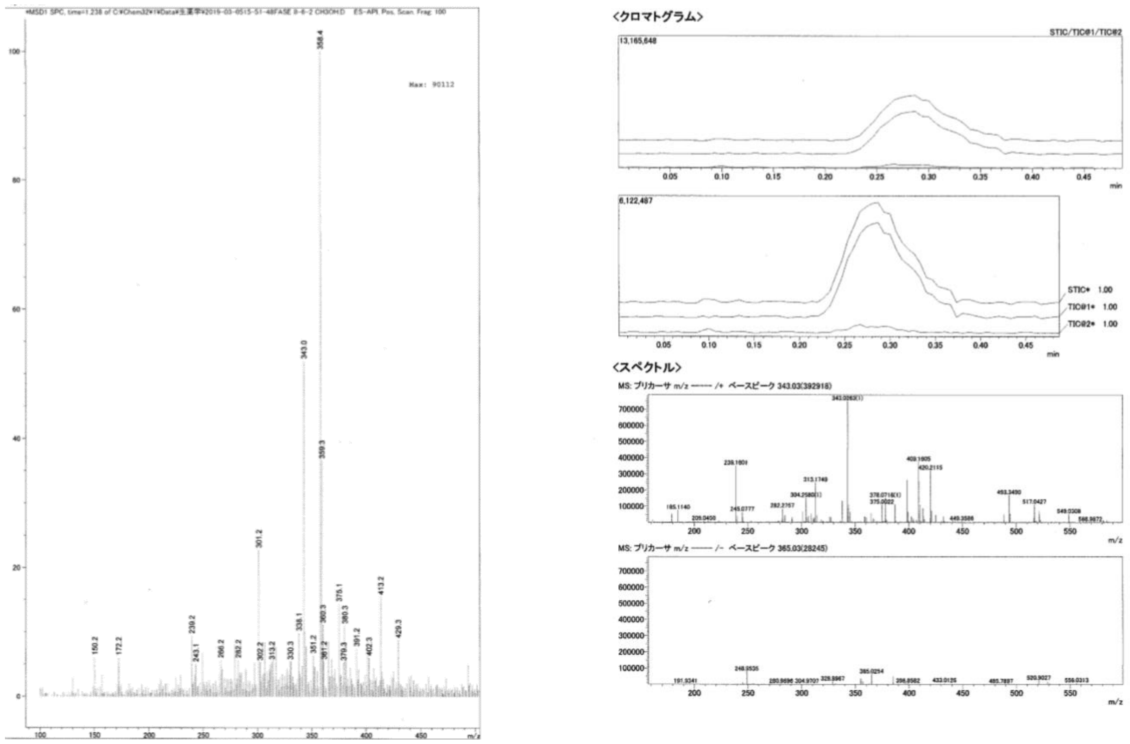

ESI-MS spectrum of compound **1**.

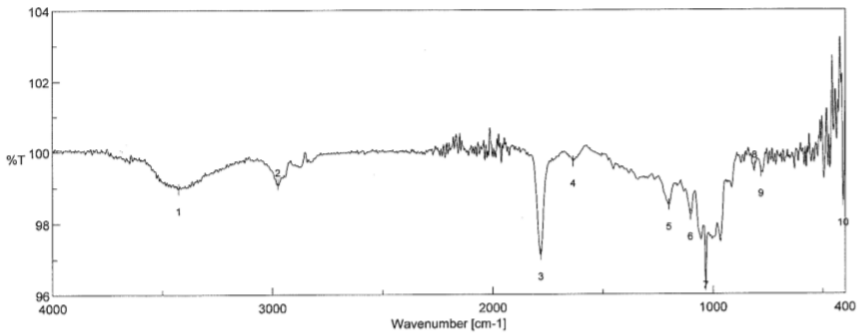

ピーク検出結果

No.	位置	強度	No.	位置	強度
1	3425.92	98.95676	2	2974.66	99.09778
3	1782.87	97.11527	4	1636.3	99.71408
5	1200.47	98.49760	6	1102.12	98.22495
7	1032.69	96.24902	8	810.92	99.56592
9	781.993	99.42392	10	409.799	98.59934

IR spectrum of compound **1**.

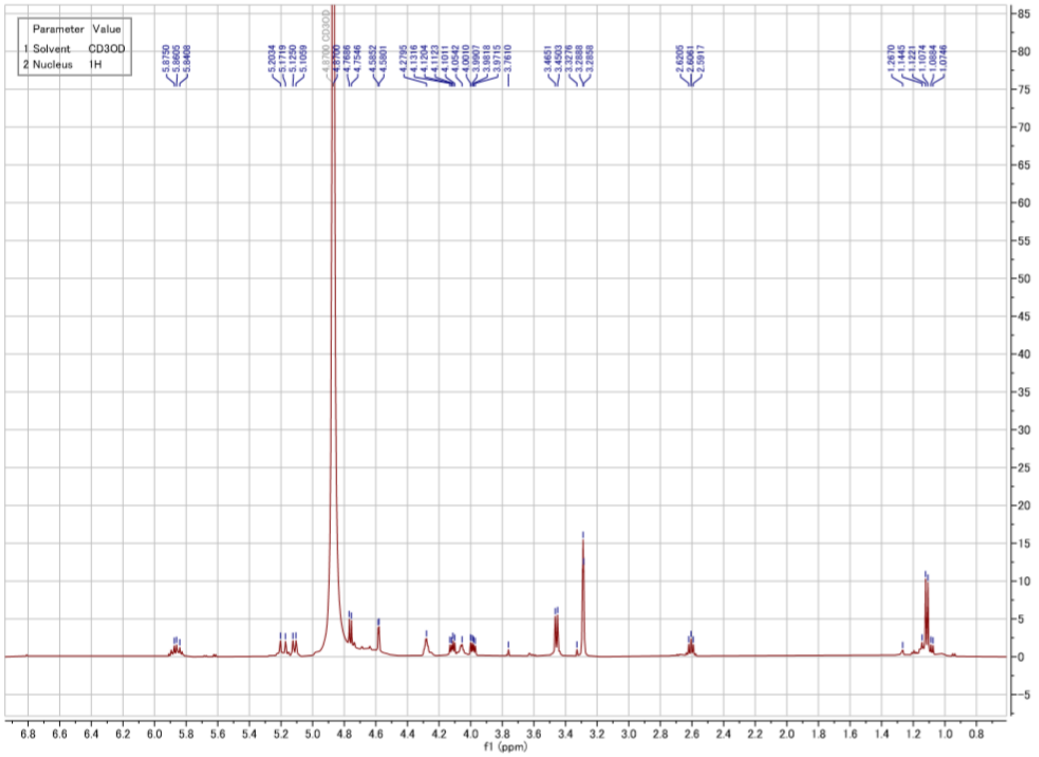

[1] H -NMR spectrum of compound **2**. Measured in CD_3OD.

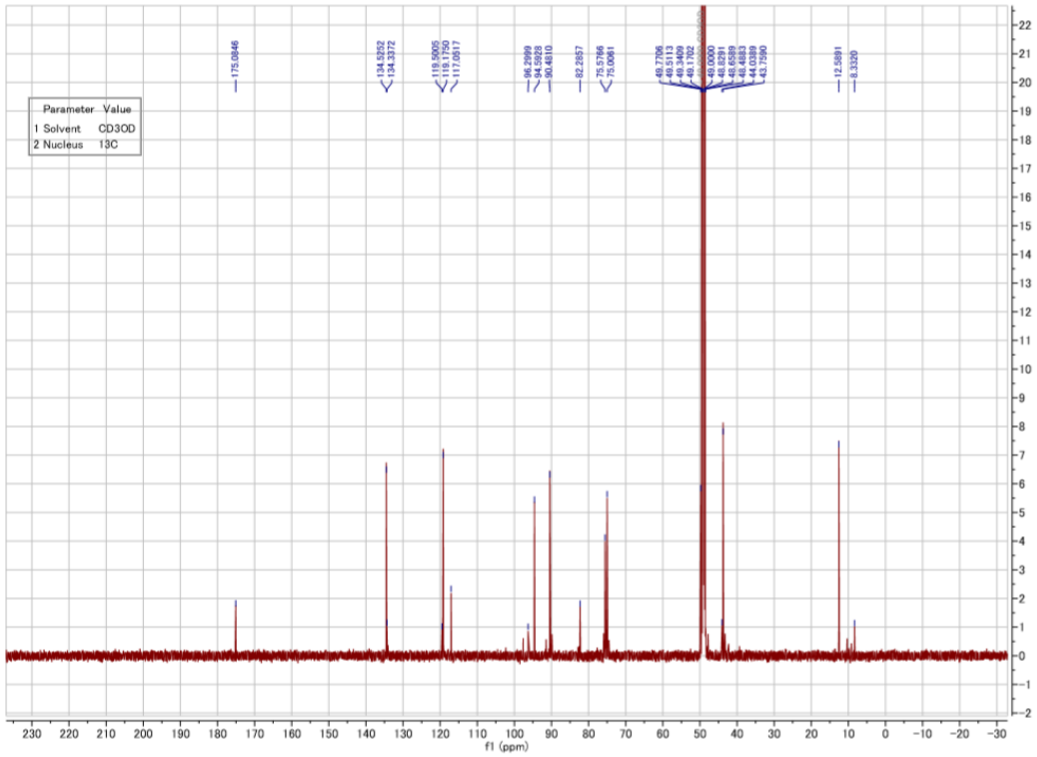

^{13}C -NMR spectrum of compound **2**. Measured in CD_3OD.

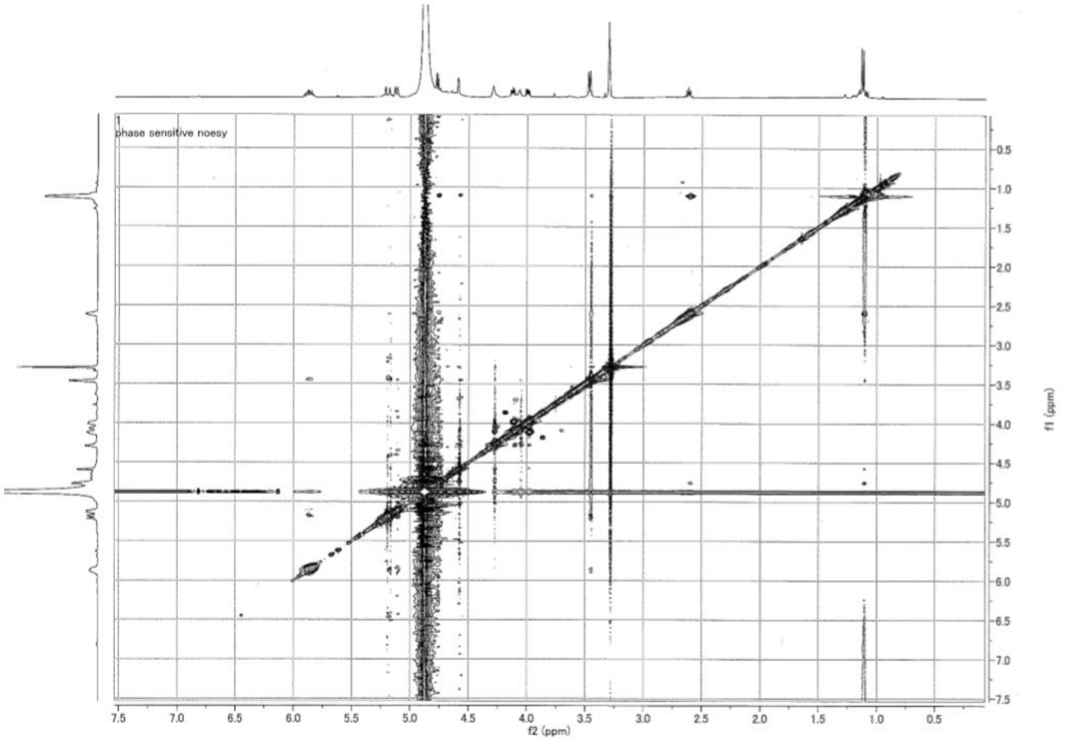

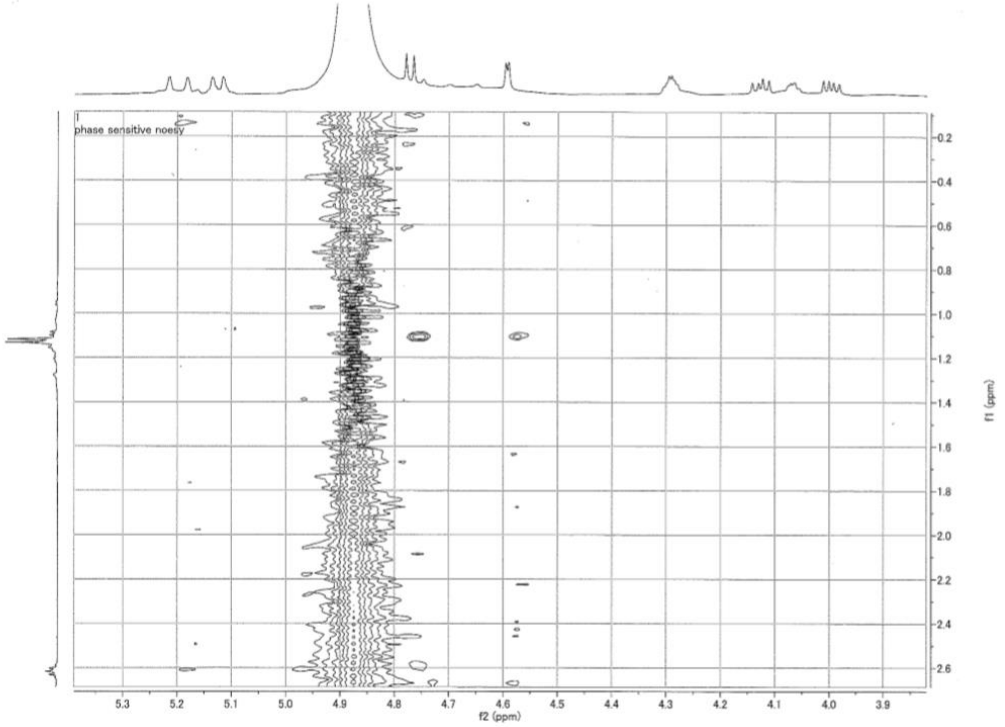

NOESY spectrum of compound **2**. Measured in CD_3OD.

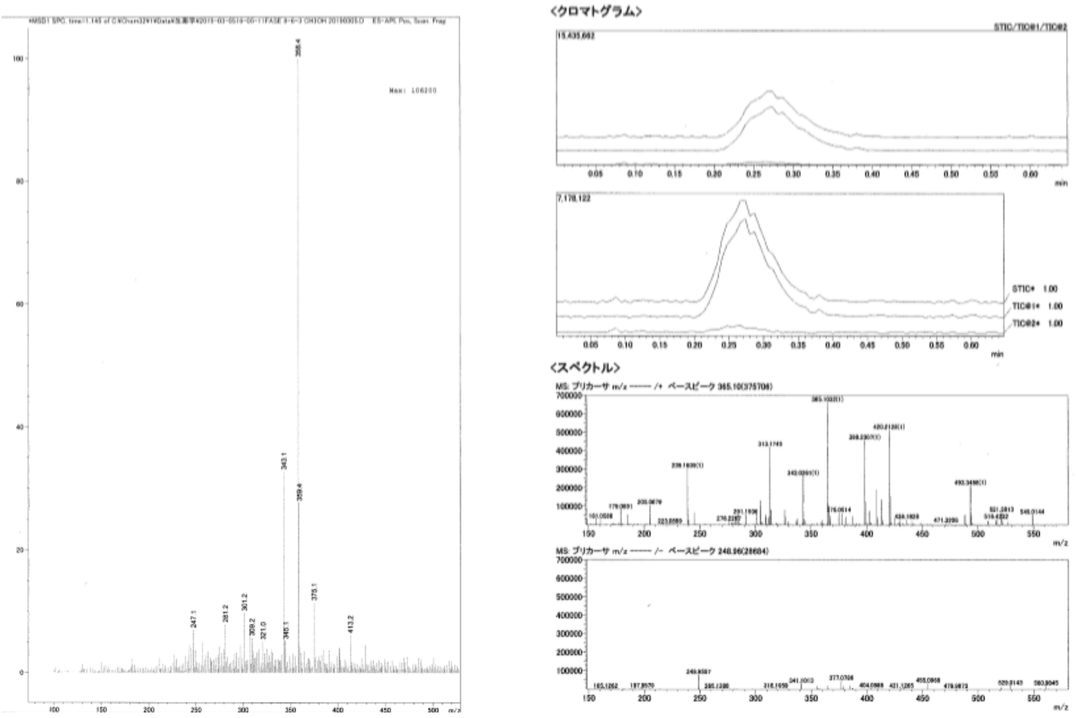

ESI-MS spectrum of compound **2**.

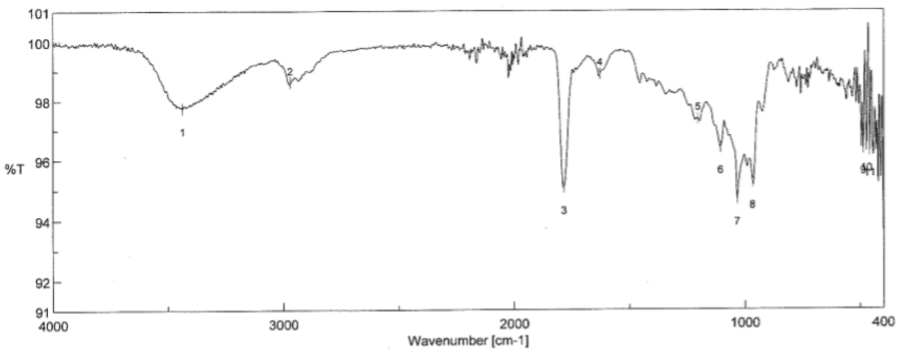

ピーク検出結果

No.	位置	強度	No.	位置	強度
1	3437.49	97.72224	2	2970.8	98.60249
3	1785.76	95.06838	4	1629.55	98.87383
5	1203.36	97.35894	6	1105.01	96.39234
7	1032.69	94.68192	8	967.126	95.22980
9	485.009	96.35419	10	470.546	96.44712
11	457.047	96.27924			

IR spectrum of compound **2**.

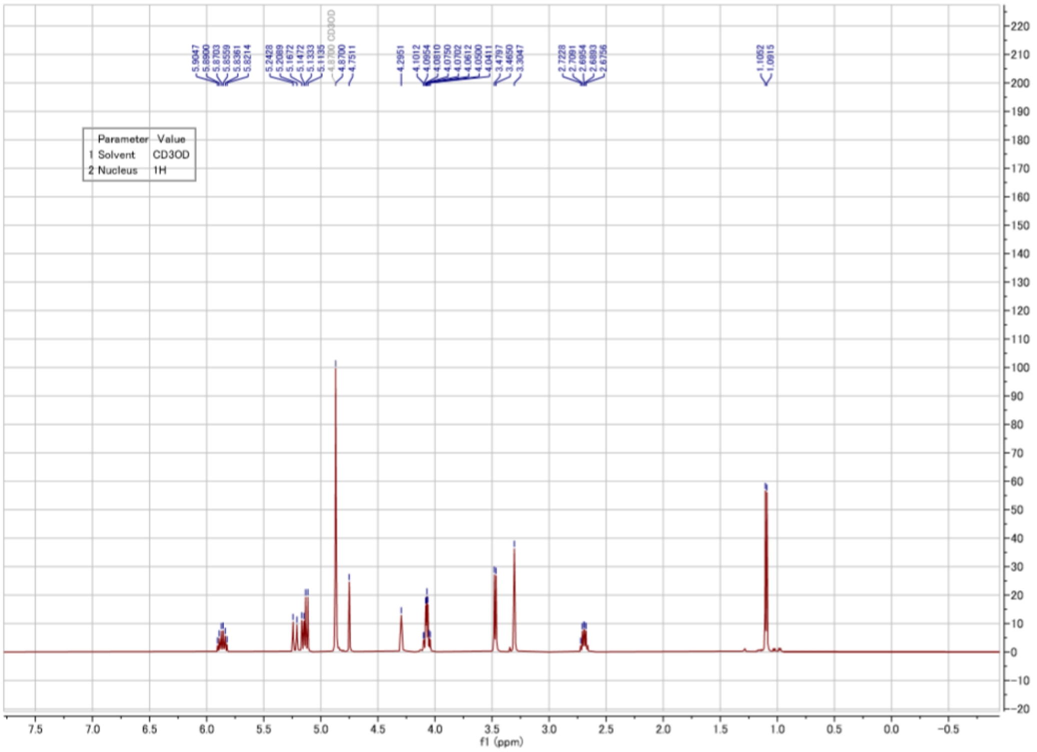

^{1}H -NMR spectrum of compound **3**. Measured in CD_3OD.

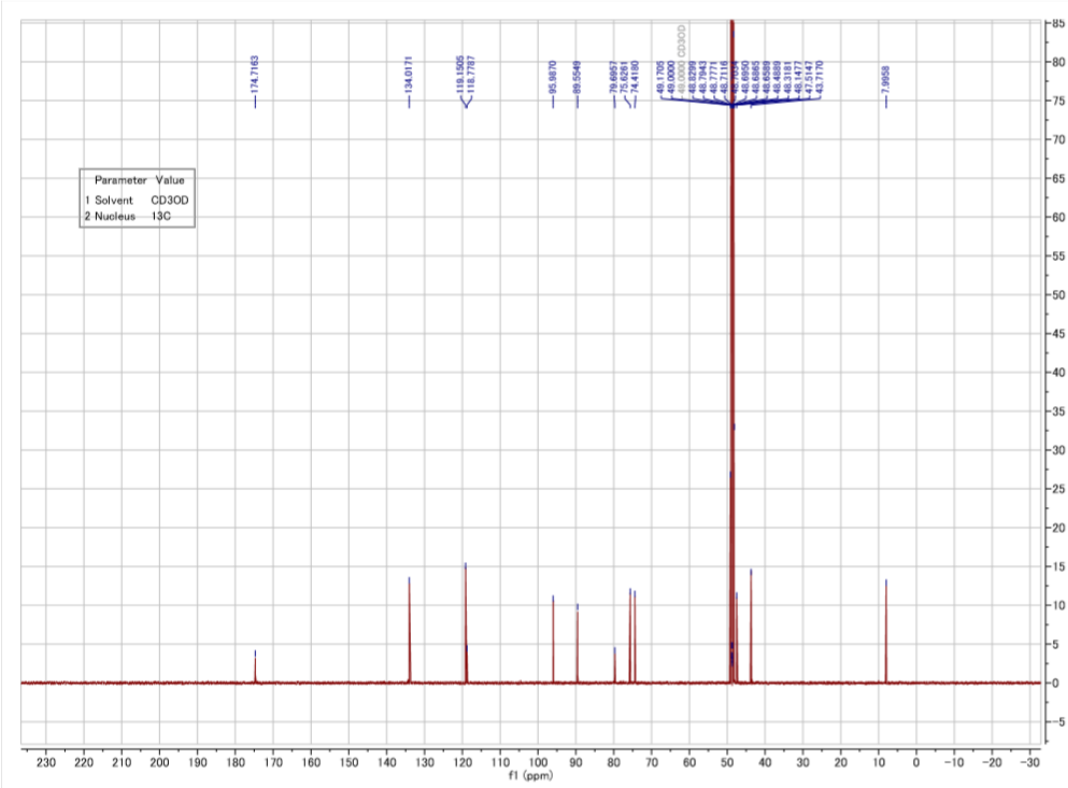

^{13}C -NMR spectrum of compound **3**. Measured in CD_3OD.

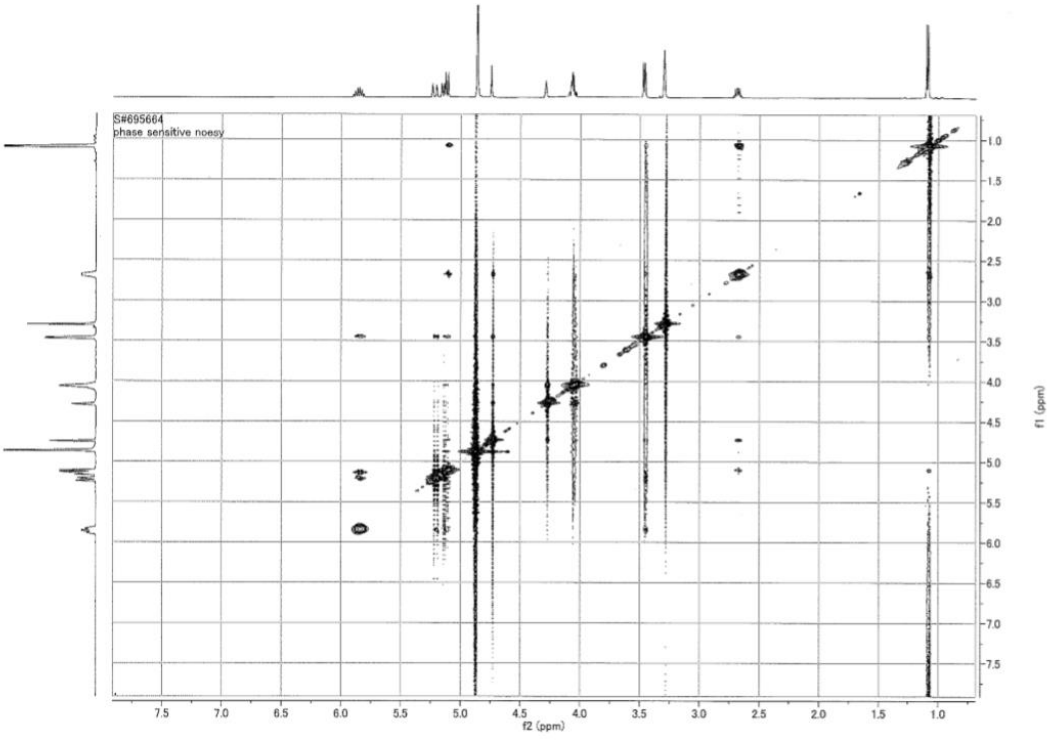
S#695664
phase sensitive noesy
f2 (ppm)
f1 (ppm)

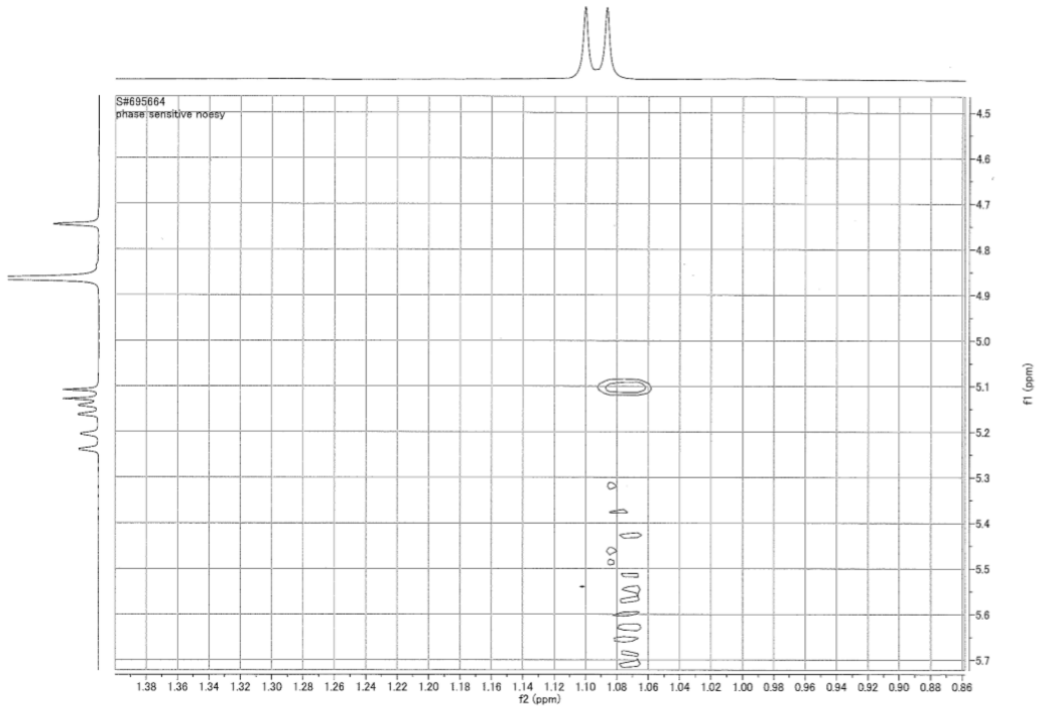
S#695664
phase sensitive noesy
f2 (ppm)
f1 (ppm)

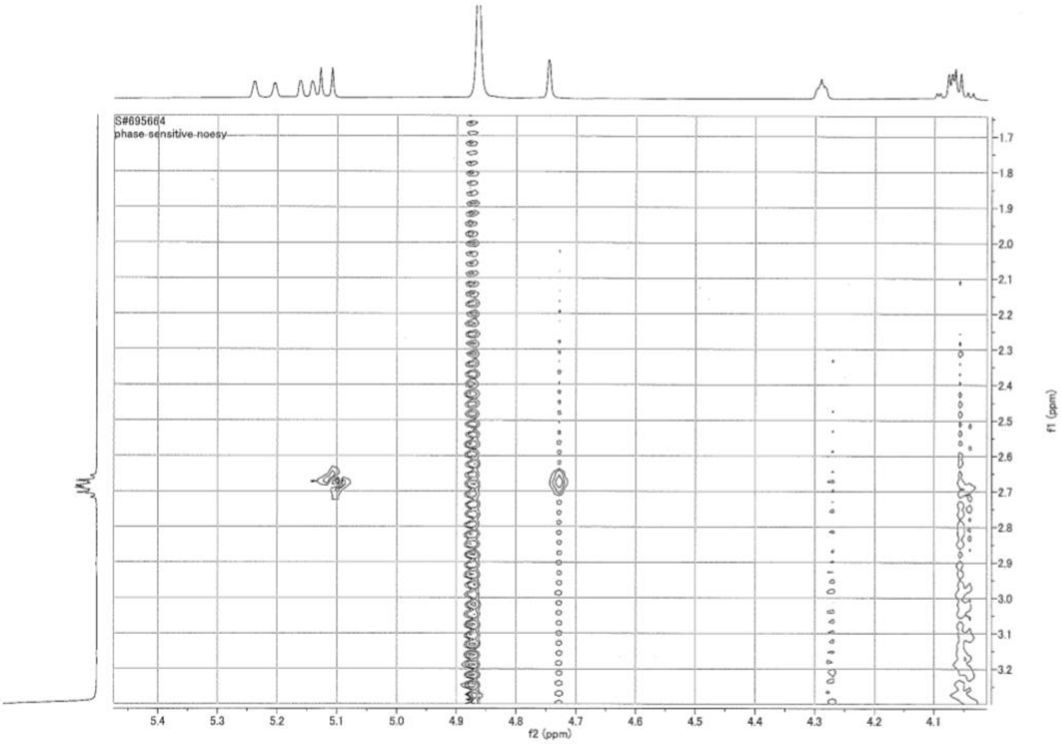

NOESY spectrum of compound **3**. Measured in CD_3OD.

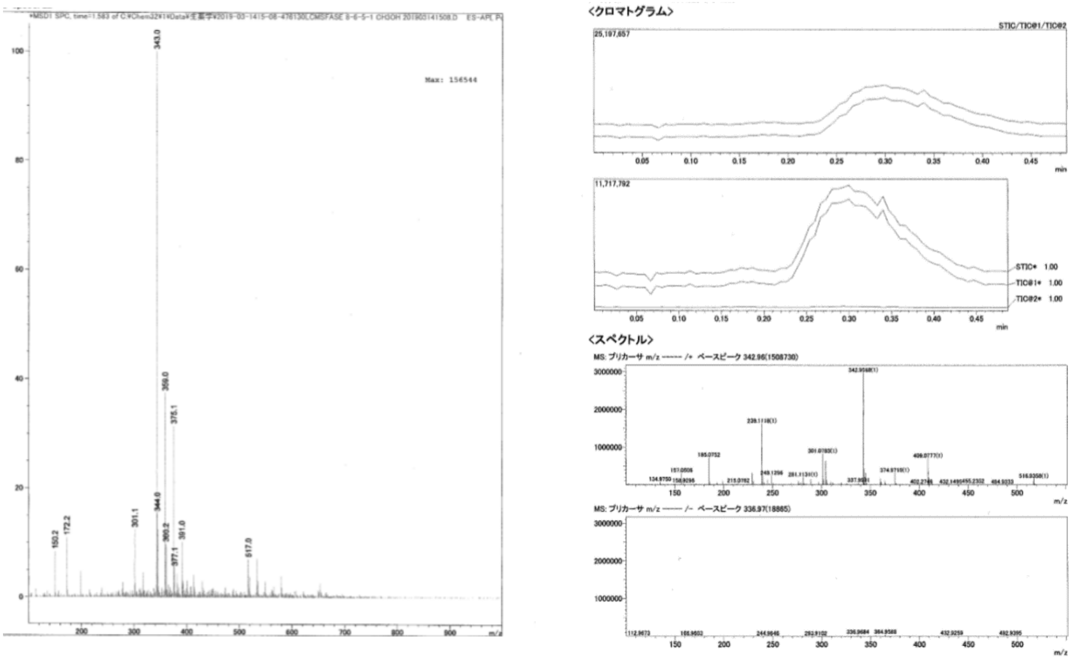

ESI-MS spectrum of compound **3**.

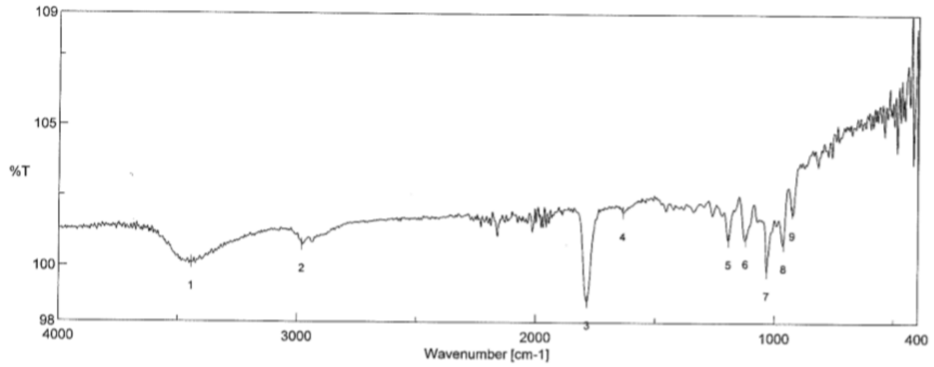

ピーク検出結果

No.	位置	強度	No.	位置	強度
1	3443.28	100.10980	2	2981.41	100.75142
3	1785.76	98.76659	4	1636.3	101.91030
5	1192.76	100.94292	6	1120.44	100.95784
7	1032.69	99.83585	8	963.269	100.80225
9	927.593	101.95715			

IR spectrum of compound **3**.

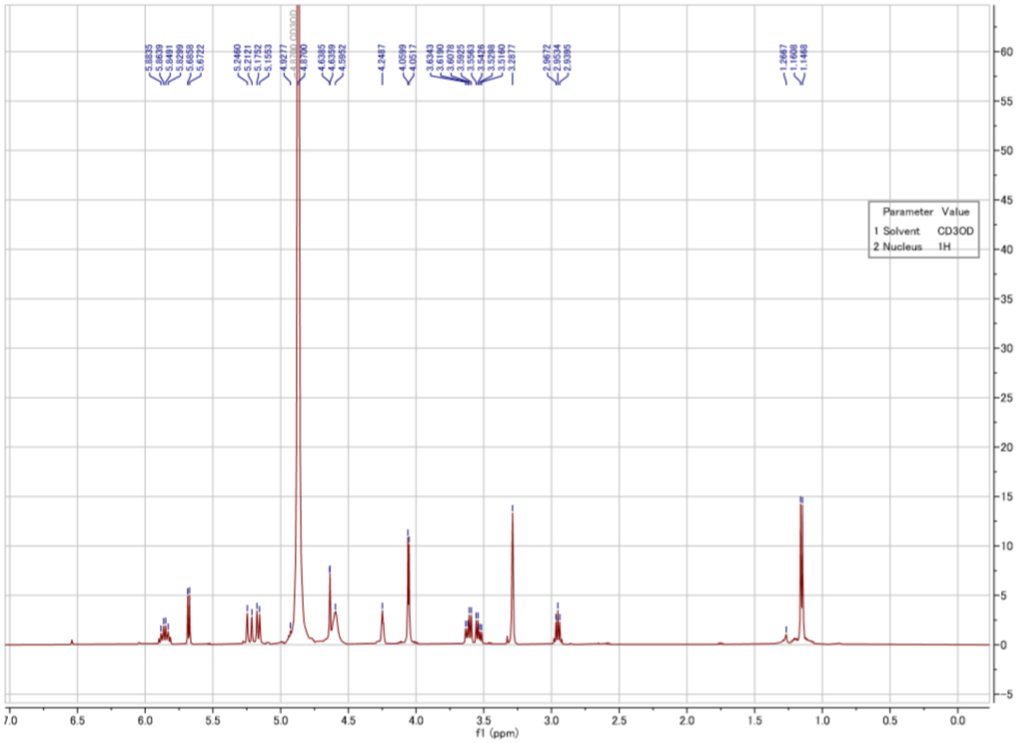

^{1}H -NMR spectrum of compound **4**. Measured in CD_3OD.

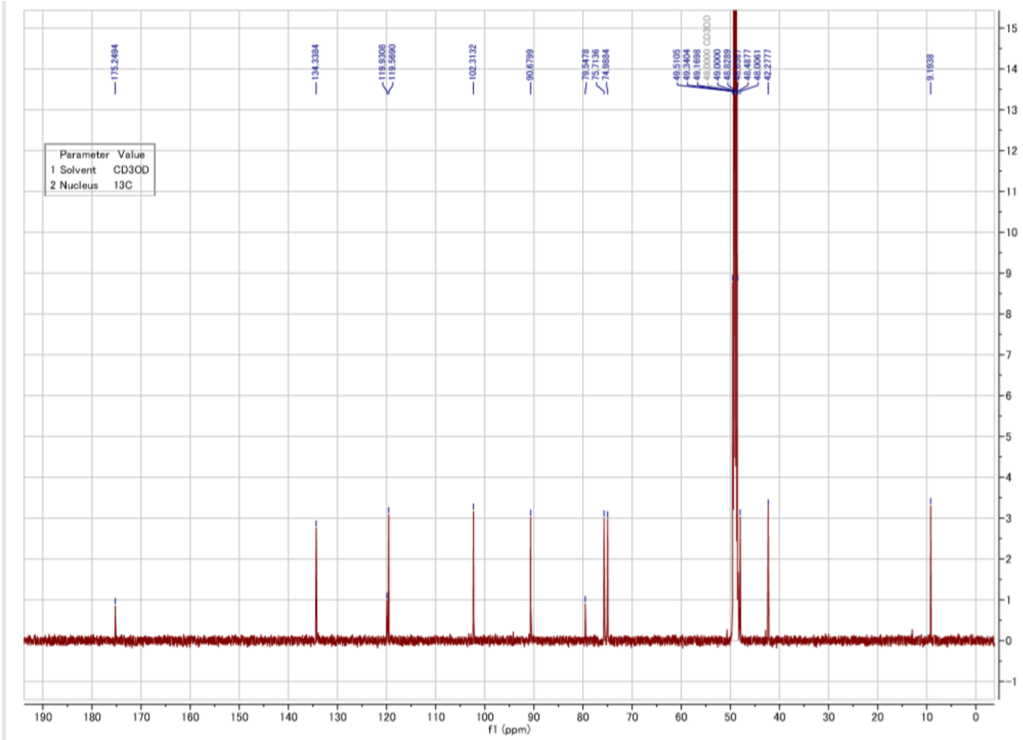

^{13}C -NMR spectrum of compound **4**. Measured in CD_3OD.

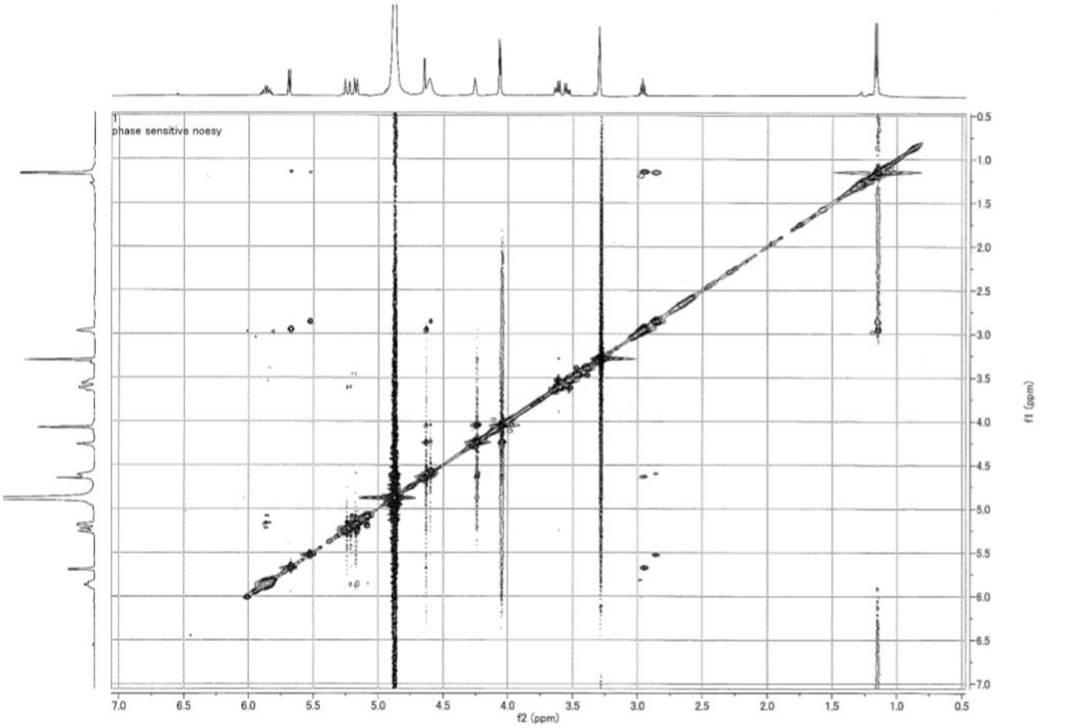
phase sensitive noesy
f2 (ppm)
f1 (ppm)

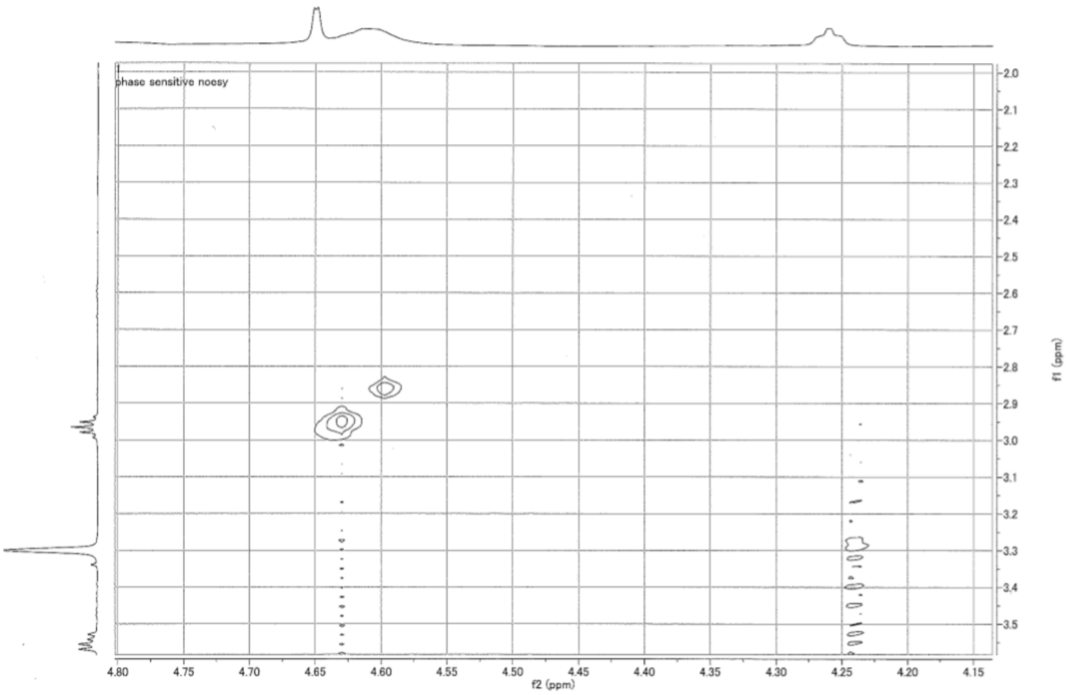
phase sensitive noesy
f2 (ppm)
f1 (ppm)

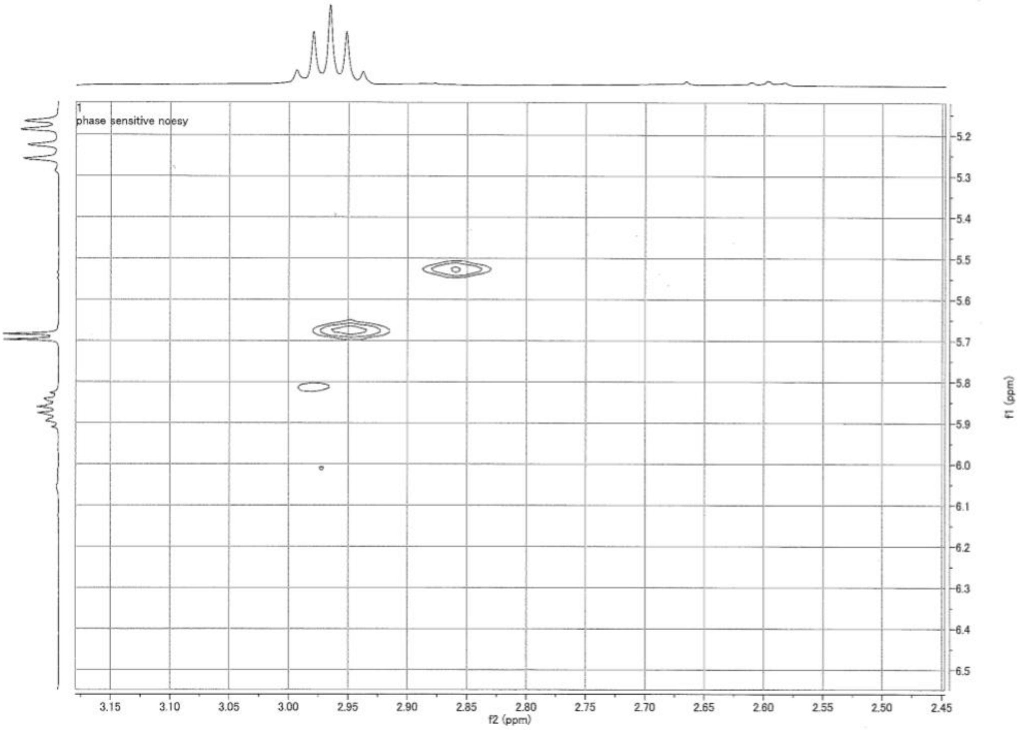

NOESY spectrum of compound **4**. Measured in CD_3OD.

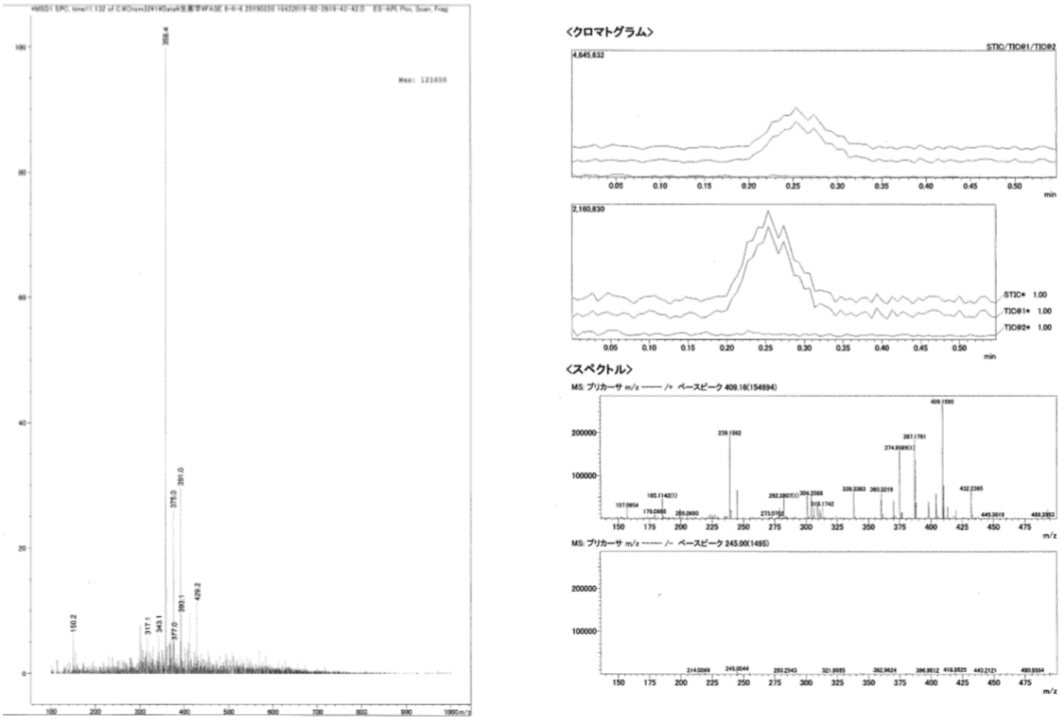

ESI-MS spectrum of compound **4**.

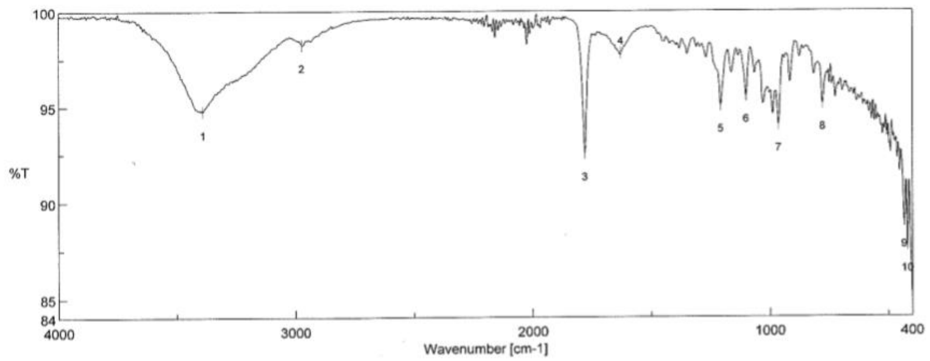

ピーク検出結果

No.	位置	強度	No.	位置	強度
1	3390.24	94.72341	2	2974.66	98.23774
3	1780.94	92.51196	4	1629.55	97.80992
5	1209.15	95.01817	6	1101.15	95.51522
7	964.233	93.99761	8	780.065	95.11114
9	432.941	88.98573	10	420.406	87.73071

IR spectrum of compound **4**.

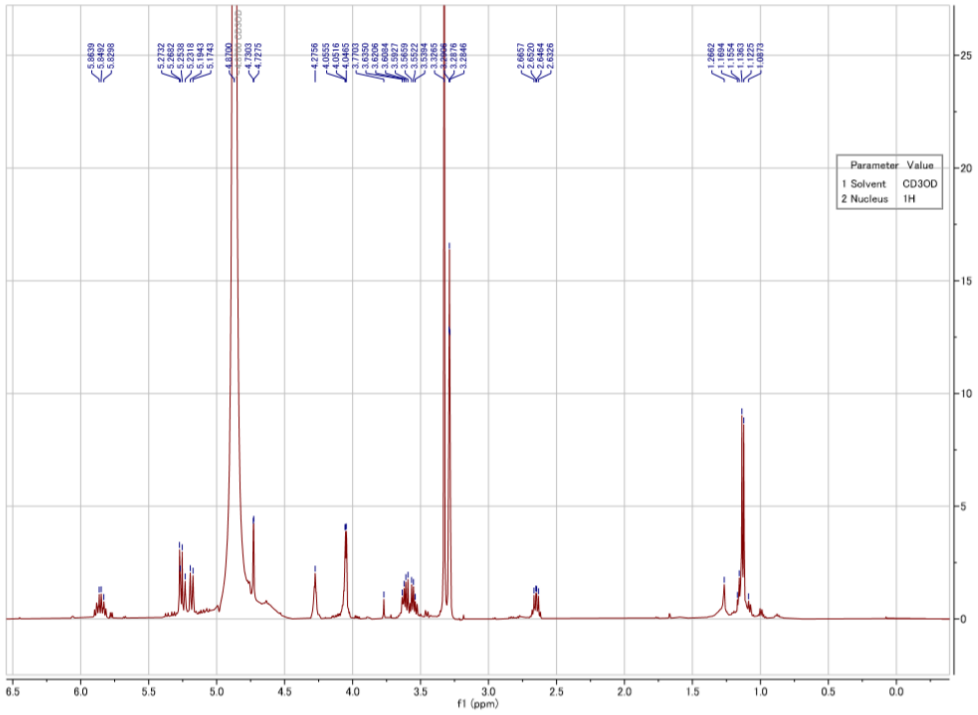

^{1}H -NMR spectrum of compound **5**. Measured in CD_3OD.

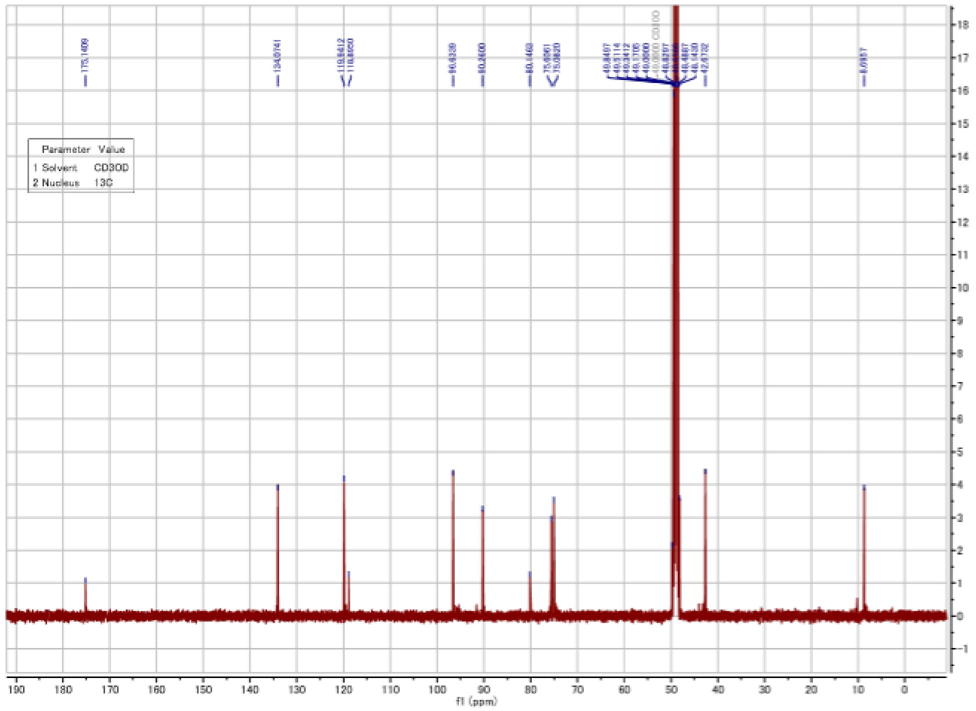

^{13}C -NMR spectrum of compound **5**. Measured in CD_3OD.

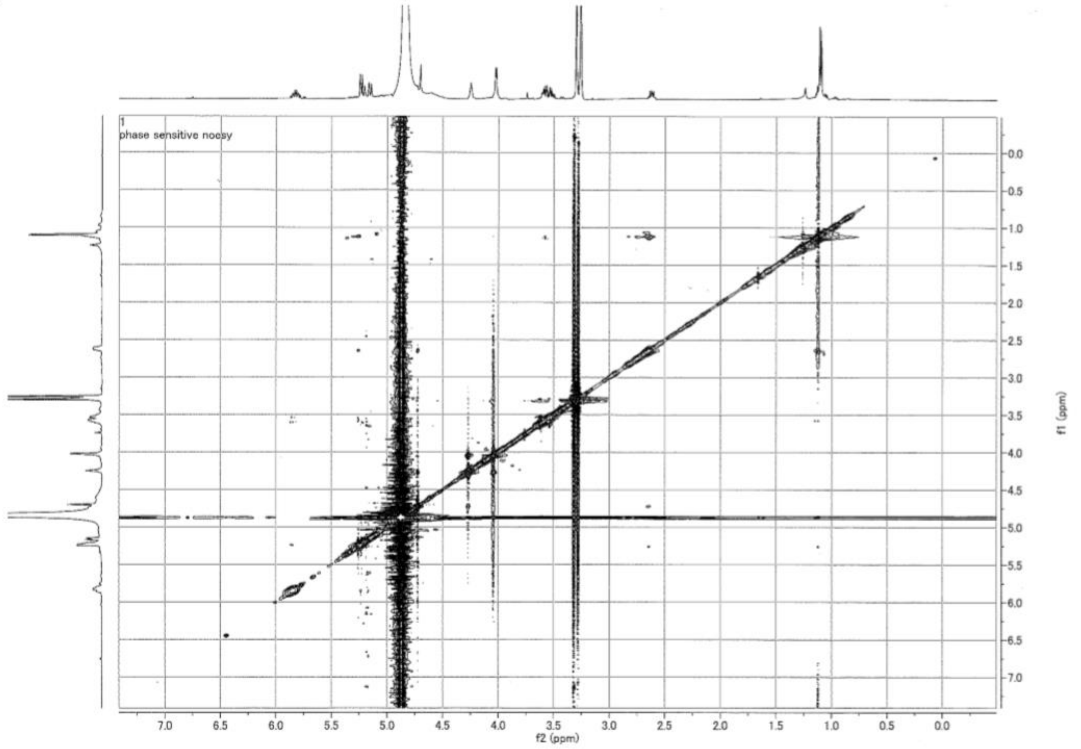
phase sensitive noesy
f2 (ppm)
f1 (ppm)

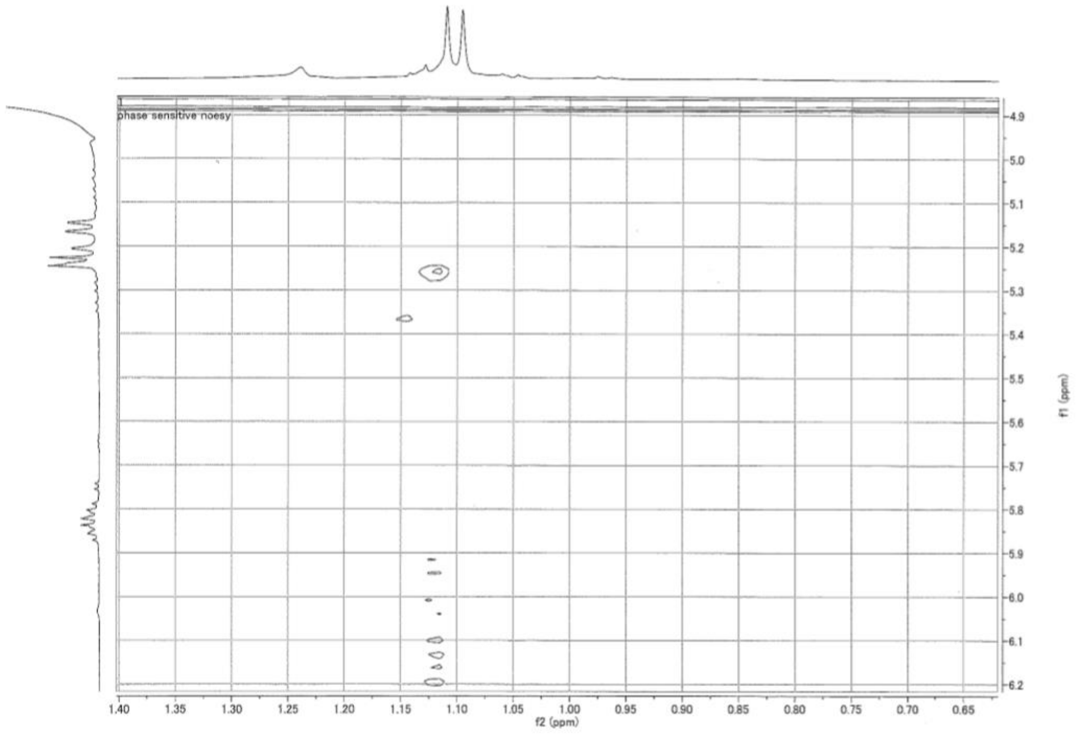
phase sensitive noesy
f2 (ppm)
f1 (ppm)

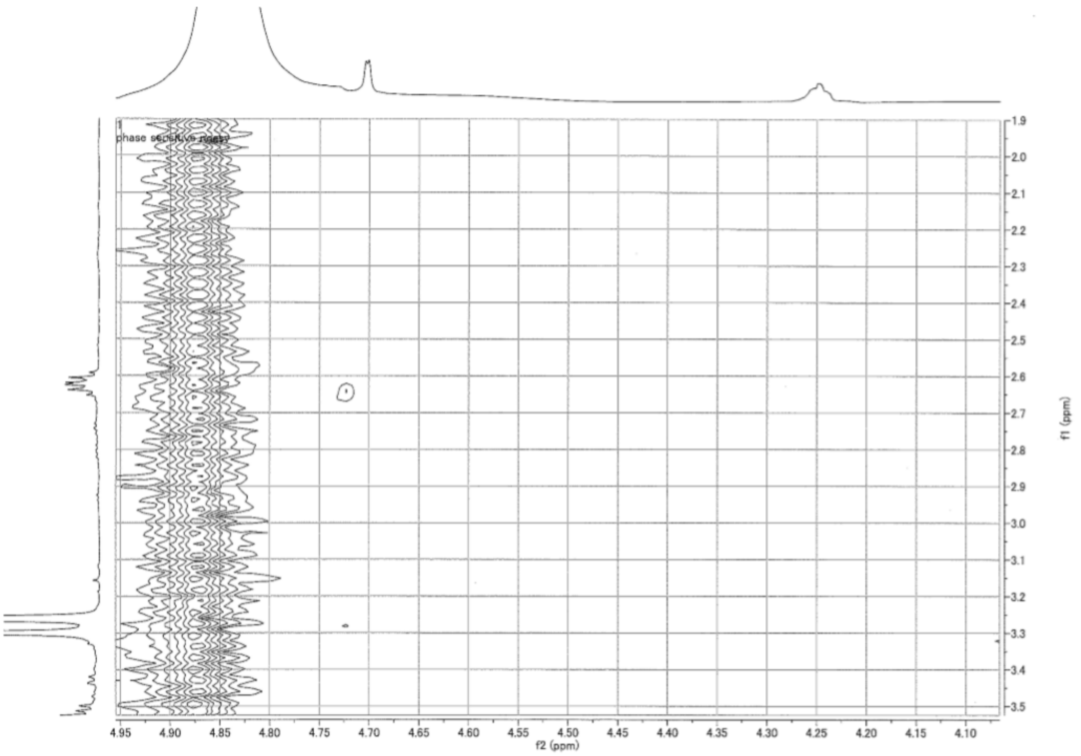

NOESY spectrum of compound **5**. Measured in CD_3OD.

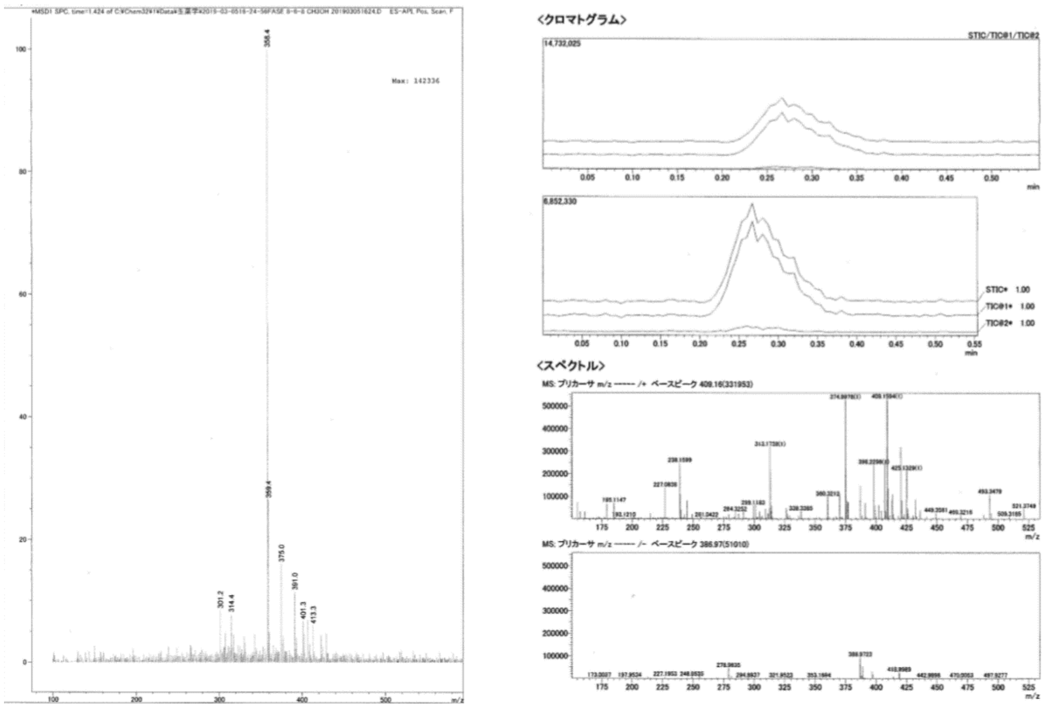

ESI-MS spectrum of compound **5**.

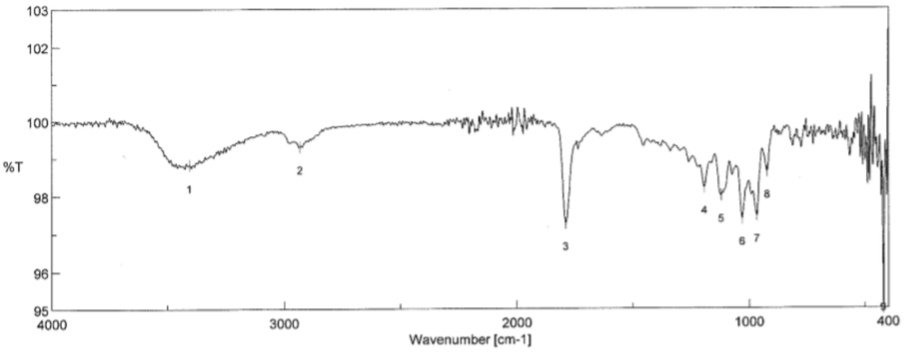

ピーク検出結果

No.	位置	強度	No.	位置	強度
1	3407.6	98.81552	2	2931.27	99.29865
3	1789.62	97.26624	4	1192.76	98.21162
5	1120.44	97.98720	6	1029.8	97.38039
7	967.126	97.45602	8	923.736	98.62030
9	421.37	95.00594			

IR spectrum of compound **5**.
